# Transcriptomic analysis reveals sex-specific patterns in the hippocampus in Alzheimer’s disease

**DOI:** 10.3389/fendo.2024.1345498

**Published:** 2024-04-16

**Authors:** Anna Onisiforou, Christiana C. Christodoulou, Eleni Zamba-Papanicolaou, Panos Zanos, Polymnia Georgiou

**Affiliations:** ^1^ Translational Neuropharmacology Laboratory, Department of Psychology, University of Cyprus, Nicosia, Cyprus; ^2^ Neuroepidemiology Department, The Cyprus Institute of Neurology and Genetics, Nicosia, Cyprus; ^3^ Laboratory of Epigenetics and Gene Regulation, Department of Biological Sciences, University of Cyprus, Nicosia, Cyprus; ^4^ Psychoneuroendocrinology Laboratory, Department of Psychology, University of Wisconsin Milwaukee, Milwaukee, WI, United States

**Keywords:** Alzheimer’s disease, hippocampus, sex-specific, females, males, estrogen, estrogen receptors

## Abstract

**Background:**

The hippocampus, vital for memory and learning, is among the first brain regions affected in Alzheimer’s Disease (AD) and exhibits adult neurogenesis. Women face twice the risk of developing AD compare to men, making it crucial to understand sex differences in hippocampal function for comprehending AD susceptibility.

**Methods:**

We conducted a comprehensive analysis of bulk mRNA postmortem samples from the whole hippocampus (GSE48350, GSE5281) and its CA1 and CA3 subfields (GSE29378). Our aim was to perform a comparative molecular signatures analysis, investigating sex-specific differences and similarities in the hippocampus and its subfields in AD. This involved comparing the gene expression profiles among: (a) male controls (M-controls) vs. female controls (F-controls), (b) females with AD (F-AD) vs. F-controls, (c) males with AD (M-AD) vs. M-controls, and (d) M-AD vs. F-AD. Furthermore, we identified AD susceptibility genes interacting with key targets of menopause hormone replacement drugs, specifically the *ESR1* and *ESR2* genes, along with *GPER1*.

**Results:**

The hippocampal analysis revealed contrasting patterns between M-AD vs. M-controls and F-AD vs. F-controls, as well as M-controls vs. F-controls. Notably, *BACE1*, a key enzyme linked to amyloid-beta production in AD pathology, was found to be upregulated in M-controls compared to F-controls in both CA1 and CA3 hippocampal subfields. In M-AD vs. M-controls, the GABAergic synapse was downregulated, and the Estrogen signaling pathway was upregulated in both subfields, unlike in F-AD vs. F-controls. Analysis of the whole hippocampus also revealed upregulation of the GABAergic synapse in F-AD vs. F-controls. While direct comparison of M-AD vs. F-AD, revealed a small upregulation of the *ESR1* gene in the CA1 subfield of males. Conversely, F-AD vs. F-controls exhibited downregulation of the Dopaminergic synapse in both subfields, while the Calcium signaling pathway showed mixed regulation, being upregulated in CA1 but downregulated in CA3, unlike in M-AD vs. M-controls. The upregulated Estrogen signaling pathway in M-AD, suggests a compensatory response to neurodegenerative specifically in males with AD. Our results also identified potential susceptibility genes interacting with *ESR1* and *ESR2*, including *MAPK1, IGF1, AKT1, TP53* and *CD44*.

**Conclusion:**

These findings underscore the importance of sex-specific disease mechanisms in AD pathogenesis. Region-specific analysis offers a more detailed examination of localized changes in the hippocampus, enabling to capture sex-specific molecular patterns in AD susceptibility and progression.

## 1 Introduction

Alzheimer’s disease (AD) is a neurodegenerative condition that significantly impacts memory, cognition and behavior, leading to dementia. Predominantly affecting individuals over 65, AD also presents in a small percentage as early-onset (EAOD), occurring before 65, and accounting for approximately 5% to 6% of all AD cases (1). Late-onset AD (LOAD) or sporadic AD is more common, manifesting later in life (onset after 65 years of age) and comprising approximately 95% of AD cases (2).The exact cause of AD is unknown, but it likely involves a mixed combination of genetic, environmental and lifestyle factors (3). AD is characterized by the accumulation of amyloid-beta (Aβ) plaques in the brain, which are derived from the cleavage of amyloid precursor protein (APP) (4). Another key pathological hallmark of AD is the formation of neurofibrillary tangles (NFTs) composed of hyperphosphorylated tau protein. Both Aβ and NFTs contribute to neuroinflammation, synaptic dysfunction, and neuronal degeneration in the brain (5, 6). Currently, there is no effective pharmacotherapy for AD, underscoring the importance to enhance our mechanistic understanding of AD for the development of novel and more effective interventions.

Neuroimaging studies consistently reveal the hippocampus, crucial for memory formation and learning, as one of the first brain region affected in AD (7). Notably, it is one of the few brain regions that exhibits adult neurogenesis. Women constituting two-thirds of AD cases, experience greater cognitive decline than men (8, 9). In the prodromal stage of AD, females with Mild Cognitive Impairment (MCI) display more pronounced cognitive decline than males (10). Neuroimaging suggests a more significant impact of hippocampal deterioration in females with AD, with higher hippocampal volume indicating reduced AD risk in women (11). Sex differences in AD may be linked to gonadal hormones, particularly estrogen. This predominantly female hormone plays a neuroprotective role in the hippocampus through various mechanisms, including the modulation of synaptic plasticity and the reduction of neuroinflammation, ultimately enhancing learning and memory (12). With women exhibiting biological variabilities compared to men, including hormonal influences and immune responses (13), understanding sex differences in the hippocampus is crucial for comprehending AD susceptibility in women.

Estrogen therapy in postmenopausal women, used to alleviate symptoms of menopause, has the potential to delay the onset of AD (14). Supported by animal studies showing estrogen’s ability to reduce Aβ production and tau hyperphosphorylation (15), recent evidence indicates that early menopause occurring between 40-45 years old and late initiation of hormone therapy are associated with higher levels of tau vulnerability, especially when Aβ is elevated (16). Females exhibit higher tau levels than age-matched controlled males (16). Genome-wide analysis has demonstrated a striking overlap between genes upregulated by estrogen in females macaques and genes downregulated in the human postmortem AD brain, suggest that the menopausal estrogen loss may contribute to increased AD risk in women (17).

Gene polymorphism studies reveal estrogen’s role in AD risk, with polymorphisms in genes governing estrogen biosynthesis, metabolism, and signaling pathways (18). Beyond direct estrogen-related genes, other genes modulate estrogen effects on AD, forming a complex network of interactions (18). Sex-specific investigation of intronic single-nucleotide polymorphisms (SNPs) at the estrogen receptor beta (*ESR2*) gene locus indicate a noteworthy increase in AD risk in women with specific *ESR2* genotypes (19). This underscores the significance of considering sex-specific genetic factors and emphasizes the need for a deeper understanding of how polymorphisms affecting the estrogen system may contribute to unravel the mechanisms underlying AD.

In this study, we employed an integrative bioinformatics approach to identify the key molecular mechanisms through which sex differences may impact AD susceptibility in women, with a specific focus on their influence in the hippocampus. Leveraging publicly available transcriptomic data from postmortem samples of the whole hippocampus (GSE5281, GSE48350), and its CA1 and CA3 subfields (GSE29378), chosen for their provision of relevant and specific transcriptomic information, we conducted an in-depth exploration of gene expression profiles among: (a) male controls (M-controls) vs. female controls (F-controls), (b) females with AD (F-AD) vs. F-controls, (c) males with AD (M-AD) vs. M-controls, and (d) M-AD vs. F-AD. This approach sheds light on the sex-specific molecular mechanisms in the hippocampus and its subfields both under normal/baseline conditions and in the context of AD. By examining gene expression patterns across these comparisons, we aim to elucidate differences across genders in healthy individuals (normomics) and AD-specific alterations, providing valuable insights into the sex-specific molecular mechanisms underlying related to AD susceptibility. This captures region-specific alterations enhancing the ability to identify both differences and similarities in transcriptomic patterns across sexes. Finally, we identified genes associated with AD susceptibility that interact with the key targets of menopause hormone replacement drugs, specifically the *ESR1*, and *ESR2* genes, as well as *GPER1*(G protein-coupled estrogen receptor1), providing valuable insights into potential therapeutic avenues.

## 2 Methods

**Figure 1:**
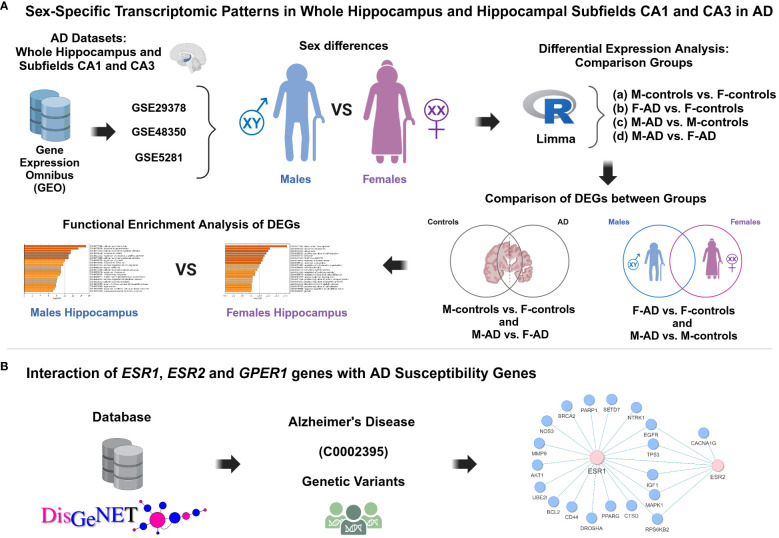
Schematic representation of the methodology applied in this study aimed to investigate the impact of sex differences on hippocampal function and AD susceptibility. **(A)** Sex-specific transcriptomic patterns in whole hippocampus and hippocampal subfields CA1 and CA3 in AD, **(B)** Interaction of *ESR1*, *ESR2* and *GPER1* genes with AD susceptibility genes.

The workflow implemented in this study is illustrated in **Figure 1**.

### 2.1 Gene expression omnibus dataset information

We extensively explored the Gene Expression Omnibus (GEO) database to identify high-throughput gene expression datasets concerning the hippocampus and AD. Our focus was on identifying transcriptomic data meeting that fulfilled specific criteria: (i) microarray expression datasets related to AD featuring hippocampal tissue; (ii) datasets with more than 4 female subjects with AD in the experimental group. Following the application of these criteria, we successfully pinpointed the transcriptomic datasets with accession numbers GSE29378 (20, 21), GSE5281 (22, 23) and GSE48350 (24, 25).

The GSE29378 (20, 21) dataset comprises postmortem brain samples from the CA1 and CA3 subfields of the hippocampus, collected from females and males patients with AD and age- and sex-matched controls, that had normal cognitive and functional examinations. In the CA1 region, the dataset includes 8 samples from F-AD, 5 samples from F-controls, 11 samples from M-controls, and 8 samples from M-AD. In the CA3 region, there are 7 samples from F-AD, 5 samples from female controls, 11 samples from M-controls, and 5 samples from M-AD. The mean average age of death of the control group is 81.7 ± 6.9, while for the AD group, the mean average age is 77.3 ± 9.1.

The GSE5281 (22, 23) dataset comprises postmortem brain samples from the hippocampus collected from patients with AD and age- and sex-matched controls, clinically classified as neurologically normal, with a mean age of death of 79.8 ± 9.1 yr. The dataset consists of 161 samples collected from six different brain regions. However, for our study only the hippocampus samples were used. The hippocampus samples comprised of 13 healthy controls and 10 AD-affected samples, including 4 F-AD and 3 F-controls, as well as 6 M-AD and 10 M-controls.

Similarly, the GSE48350 (24, 25) dataset comprises postmortem brain samples from the hippocampus collected from patients with AD (age range: 74 to 95 years) and from cognitively intact young and sex-matched controls (age range: 20 to 59 years) as well as age- and sex-matched controls (age range: 60 - 99 years). It includes 253 samples collected from four brain regions. However, for our study only the hippocampus samples were used, and from the controls, only the aged controls (60 - 99 years) were included. The hippocampus samples consisted of 26 healthy controls and 19 AD-affected samples, including 10 F-AD and 12 F-controls, as well as 9 M-AD and 14 M-controls.

**Table 1** provides a detailed description of the included datasets. Further details about the datasets can be found in the original papers (20–25).

**Table 1:** Comprehensive details of the bulk mRNA hippocampus datasets of patients with AD and controls analyzed in this study.

Accession ID	Platform	Platform Type	Sample Size (Case/Control)	Sample Type
GSE29378	GPL6947	Illumina HumanHT-12 V3.0 expression beadchip	CA1: 8 F-AD/5 F-control CA1: 8 M-AD/11 M-controls CA3: 7 F-AD/5 F-controls CA3: 8 M-AD/11 M-controls	Hippocampus CA1 and CA3 subfields
GSE5281	GPL570	Affymetrix Human Genome U133 Plus 2.0 Array	4 F-AD/3 F-controls 6 M-AD/10 M-controls	Hippocampus
GSE48350	GPL570	Affymetrix Human Genome U133 Plus 2.0 Array	10 F-AD/12 F- controls 9 M-AD/14 M-controls	Hippocampus

### 2.2 Data processing and differential gene expression analysis

The identification of differentially expressed genes (DEGs) for all three datasets (GSE29378, GSE48350, GSE5281) dataset was conducted using the Linear Models for Microarray Data (Limma) approach. Limma is an R package specifically designed for the analysis of high-throughput data, including microarray experiments, enabling the detection of DEGs (26). For the whole hippocampus (GSE48350, GSE5281) and each of the two hippocampal subfields, CA1 and CA3 (GSE29378), differential expression analysis was conducted among: (a) M-controls vs. F-controls, (b) F-AD vs. F-controls, (c) M-AD vs. M-controls, and (d) M-AD vs. F-AD.

Comparing M-controls vs. F-controls (normomics) is crucial to understand baseline sex differences in gene expression between healthy controls with normal cognitive and functional examinations. Additionally, comparing F-AD vs. F-controls and M-AD vs. M-controls provides insights into gene expression changes specific to AD within each sex, helping identify sex-specific molecular signatures of the disease. Conversely, comparing M-AD vs. F-AD directly assesses sex-specific differences in AD pathology, revealing gene expression alterations that may contribute to sex disparities in AD susceptibility and progression.

The GSE29378, GSE48350 and GSE5281 datasets were normalized and log_2_ transformed. Following the Limma analysis, the top 400 DEGs (top 200 upregulated and top 200 downregulated DEGs) with uncorrected *p*-value of <0.05 were selected for each of the three datasets for further analysis. For the GSE29378 dataset very few genes showed significant differential expression when adjusted p-value cutoff of <0.05 was used; thus, a more lenient cutoff of threshold was selected. This decision is consistent with the original paper that provided the datasets, where they also used uncorrected *p*-value of <0.05 (20). For, consistency uncorrected *p*-value of <0.05, was also used for the other two datasets (GSE48350 and GSE5281). For the number of genes that survive using more stringent cut-off values including an uncorrected p <0.01, adjusted p <0.05 and adjusted p<0.01 for each dataset see **Supplementary Table S1.**

The selection of the top 400 DEGs aimed to focus on a subset with the most significant changes in expression, capturing key molecular alterations associated with AD in the hippocampus and its subfields, to be used for enrichment analysis, as analyzing the entire set of DEGs would have been challenging to interpret.

Moreover, to further explore gene differences between the comparison groups, we identified the opposite differentially expressed genes (DEGs) for all three datasets between (a) F-AD vs. F-controls and M-AD vs. M-controls, and (b) M-controls vs. F-controls and M-AD vs. F-AD by calculating the absolute difference in logFC.

### 2.3 Functional enrichment analysis of the DEGs from the whole hippocampus and hippocampal CA1 and CA3 subfields datasets

Enrichment analysis was performed using Metascape, an open-access enrichment analysis web tool (27). The following parameters were selected: a p-value < 0.01, minimum overlap set to 3, and a minimum enrichment factor (the ratio of observed count to chance-expected counts) set to >1.5. P-values were determined using the cumulative hypergeometric distribution, and q-values were derived using the Benjamini-Hochberg method. Terms meeting these criteria were grouped into clusters based on their membership similarity, and the most statistically significant term in each cluster was selected for representation (27). The intensity of the orange color corresponds to the p-value of the term. A darker shade indicates a more significant p-value.

Kappa Scores were used as the similarity metric for hierarchical clustering on the enriched terms obtained. Sub-trees with a similarity exceeding 0.3 were then grouped together. For the enrichment analysis, the organism Homo sapiens was selected, and the Kyoto Encyclopedia of Genes and Genomes (KEGG) library and Gene Ontology (GO) library for Biological Processes (BP) were utilized. To ensure a more focused analysis on nervous system-related processes, cancer pathways were excluded from the KEGG pathway results. This refinement aims to enhance the specificity of the analysis within the context of the nervous system.

Enrichment analysis was first performed using the top 200 upregulated and top 200 downregulated DEGs together, for both the KEGG and GO BP databases. This approach provides a broad perspective on dysregulated pathways and biological processes associated with AD in males and females, without bias towards either upregulated or downregulated genes. By analyzing all DEGs together, we gain insights into the overall molecular landscape and identify pathways/processes that are globally perturbed in AD.

Subsequently, we performed separate enrichment analyses for the top 200 upregulated and top 200 downregulated DEGs to discern the regulatory direction of the enriched KEGG pathways derived from the combined DEG sets. This focused approach allows to pinpoint pathways that are selectively dysregulated in one direction, thus offering insight into the specific molecular alterations driving AD pathology across sexes.

### 2.4 Interaction of *ESR1*, *ESR2* and *GPER1* with AD susceptibility genes

DisGeNET database (28) was utilized to collect genetic susceptibility genes associated with AD (C0002395). After eliminating duplicate entries, a total of 766 gene names were collected. Subsequently, these gene names were used as input for the String: protein app in Cytoscape (29) to construct a PPI network encompassing the variant-AD associations. Among the 766 collected gene names, 518 were identified within the *String: protein app*. A confidence cut-off score of 0.7 was set for the PPIs, with scores ranging from 0 to 1.0, denoting low to high confidence in the interactions. A higher score indicates a greater likelihood that the PPIs are true positives (30), and a cut-off of 0.7 or higher is recommended as they represent high-confidence interactions (31). The resulting AD variant-variant PPI network consisted of 518 nodes (proteins) and 1119 edge interactions. Using this reconstructed PPI network, we identified AD susceptibility genes that interact with the key targets of menopause hormone replacement drugs, specifically the *ESR1*, *ESR2* and *GPER1* genes, by identifying their first neighbors in the network.

## 3 Results

### 3.1 Sex-specific transcriptomic patterns of CA1 and CA3 hippocampal subfields in AD

In our analysis, we utilized the GSE29378 dataset, which comprises postmortem brain samples obtained from the CA1 and CA3 subfields of the hippocampus. These samples were collected from both female and male patients diagnosed with AD, as well as from controls. Our primary objective was to identify transcriptomic molecular signatures that are common between sexes and those that exhibit sex-specific patterns within these distinct hippocampal subfields. This analysis aimed to uncover sex-specific differences and commonalities in the molecular profiles of the CA1 and CA3 hippocampal subfields, providing insights into the increased susceptibility of females to AD and the underlying mechanisms contributing to sex-related differences in AD risk. For this purpose, we performed differential expression analyses in the CA1 and CA3 hippocampal subfields between: (a) M-controls vs. F-controls, (b) F-AD vs. F-controls, (c) M-AD vs. M-controls, and (d) M-AD vs. F-AD.

#### 3.1.1 DEGs of M-controls vs. F-controls

Through transcriptome comparison between M-controls vs. F-controls, we identified 2603 DEGs in the CA1 and 5403 in the CA3 hippocampal subfield (**Supplementary Tables S2, S3**).

#### 3.1.2 Comparison of F-AD vs. F-controls and M-AD vs. M-controls

Comparing F-AD vs. F-controls, we found 2247 DEGs in the CA1 and 2790 in the CA3 subfield (**Supplementary Tables S4, S5**). Similarly, comparing M-AD vs. M-controls, we identified 2247 DEGs in the CA1 and 2790 in the CA3 subfield (**Supplementary Tables S6, S7** **).**

**Figure 2:**
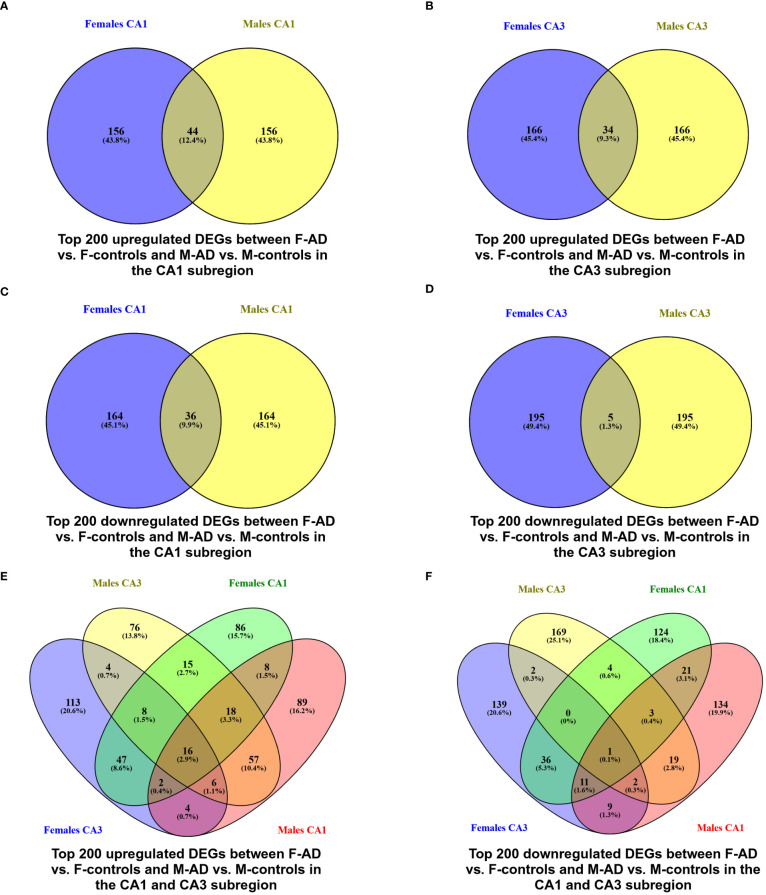
Venn Diagram indicating the common and exclusive DEGs found between F-AD vs. F-controls and M-AD vs. M-controls in the CA1 and CA3 hippocampal subfields from GSE29378 Dataset: **(A)** top 200 upregulated DEGs between F-AD vs. F-controls and M-AD vs. M-controls in the CA1 subfield, **(B)** top 200 upregulated DEGs between F-AD vs. F-controls and M-AD vs. M-controls in the CA3 subfield, **(C)** top 200 downregulated DEGs between F-AD vs. F-controls and M-AD vs. M-controls in the CA1 subfield, **(D)** top 200 downregulated DEGs between F-AD vs. F-controls and M-AD vs. M-controls in the CA3 subfield, **(E)** top 200 upregulated DEGs between F-AD vs. F-controls and M-AD vs. M-controls in both CA1 and CA3 subfields, and **(F)** top 200 downregulated DEGs between F-AD vs. F-controls and M-AD vs. M-controls in both CA1 and CA3 subfields.

Venny (https://www.biotools.fr/misc/venny) comparisons between the top 200 upregulated DEGs in F-AD vs. F-controls and M-AD vs. M-controls in the CA1 and CA3 hippocampal subfields revealed 44 and 34 common DEGs, respectively (see **Figures 2A, B**). Additionally, comparison between the top 200 downregulated DEGs in F-AD vs. F-controls and M-AD vs. M-controls in the CA1 and CA3 subfields revealed 36 and 5 common DEGs, respectively (see **Figures 2C, D**). Further analysis identified specific DEGs that are upregulated in M-AD and downregulated in F-AD, and vice versa. Notably, *SLC1A7* was upregulated in M-AD vs. M-controls and downregulated in F-AD vs. F-controls in both CA1 and CA3 subfields, while *TMEM10, MOG*, and *MAL* were upregulated in F-AD vs. F-controls and downregulated in M-AD vs. M-controls in the CA3 subfield. Additionally, the analysis results unveiled 16 common upregulated DEGs from the top 200 in both CA1 and CA3 subfields across both males and females with AD (see **Figure 2E**). These genes include *RGS1, SERPINA3, CD44, CD163, FOS, CD99, EMP1, MGST1, DTNA, NUPR1, P8, HSPB8, SPARC, C1S, YAP1*, and *ZFP36*. Notably, the *RGS1* DEG exhibited the highest log fold change (logFC) in both the CA1 and CA3 hippocampal subfields across both sexes. Additionally, the analysis revealed the *EFHD2* gene as the only common downregulated DEGs among the top 200 in both CA1 and CA3 subfields across both sexes (see **Figure 2F**).

**Figure 3:**
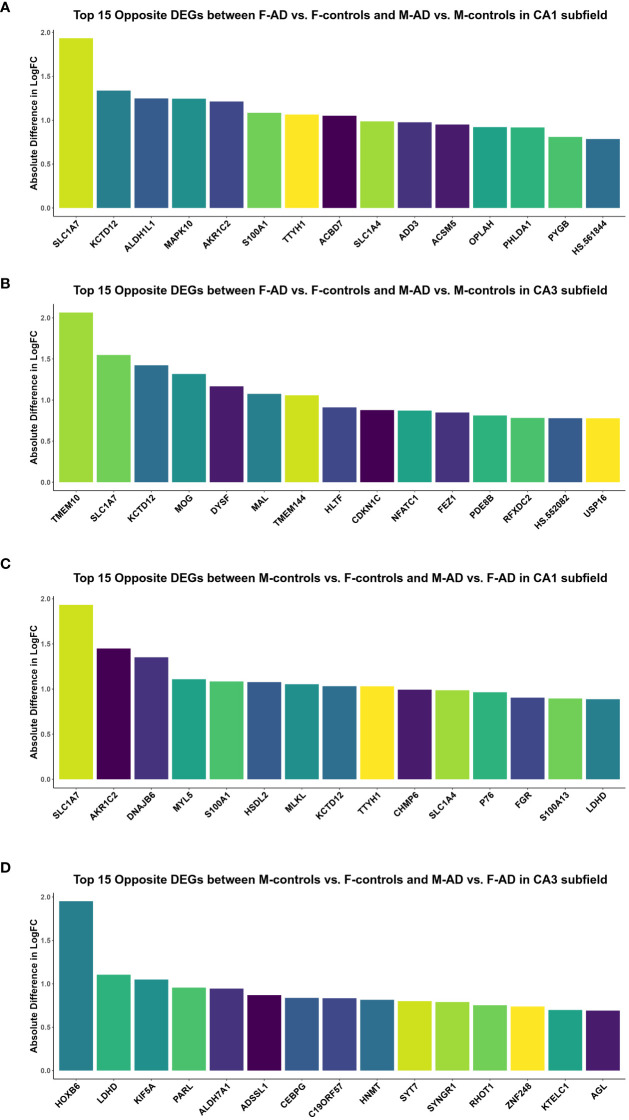
Top 15 opposite DEGs based on absolute difference in logFC between: **(A)** F-AD vs. F-controls and M-AD vs. M-controls in the CA1 subfield, **(B)** F-AD vs. F-controls and M-AD vs. M-controls in the CA3 subfield, **(C)** M-controls vs. F-controls and M-AD vs. F-AD in the CA1 subfield, and **(D)** M-controls vs. F-controls and M-AD vs. F-AD in the CA3 subfield.

Additionally, to further explore differences between F-AD vs. F-controls and M-AD vs. M-controls, we identified the opposite DEGs found between the two comparison groups in the CA1 and CA3 hippocampal subfields based on absolute difference in logFC. This approached allowed us to isolate the top 15 opposite DEGs between F-AD vs. F-controls and M-AD vs. M-controls. In the CA1 subfield, the top 15 opposite DEGs included *SLC1A7, KCTD12, ALDH1L1, MAPK10, AKR1C2, S100A1, TTYH1, ACBD7, SLC1A4, ADD3, ACSM5, OPLAH, PHLDA1, PYGB* and *HS.561844* (**Figure 3A**). Similarly, in the CA3 subfield, the top 15 opposite DEGs included *TMEM10, SLC1A7, KCTD12, MOG, DYSF, MAL, TMEM144, HLTF, CDKN1C, NFATC1, FEZ1, PDE8B, RFXDC2, HS.552082* and *USP16* (**Figure 3B**).

#### 3.1.3 DEGs of M-AD vs. F-AD

Comparing M-AD vs. F-AD, we identified 2887 DEGs in the CA1 and 1542 in the CA3 hippocampal subfield (**Supplementary Tables S8, S9**). Interestingly, the *ESR1* gene (p-value = 0.046913384, logFC = +0.127810124) was found to be upregulated in M-AD compared to F-AD in the CA1 subfield, but not in the CA3 hippocampal subfield.

Comparison between M-controls vs. F-controls and M-AD vs. F-AD based on absolute difference in logFC

To compare gene expression patterns between M-controls vs. F-controls and M-AD vs. F-AD, we identified opposite DEGs in the CA1 and CA3 hippocampal subfields based on absolute difference in logFC. This enabled to isolate the top 15 opposite DEGs between the two groups. In the CA1 subfield, these DEGs included *SLC1A7, AKR1C2, DNAJB6, MYL5, S100A1, HSDL2, MLKL, KCTD12, TTYH1, CHMP6, SLC1A4, P76, FGR, S100A13*, and *LDHD* (**Figure 3C**). Conversely, in the CA3 subfield, the top 15 opposite DEGs consisted of *HOXB6, LDHD, KIF5A, PARL, ALDH7A1, ADSSL1, CEBPG, C19ORF57, HNMT, SYT7, SYNGR1, RHOT1, ZNF248, KTELC1* and *AGL* (**Figure 3D**).

### 3.2 KEGG and GO enrichment analysis of shared DEGs and sex-specific patterns in the CA1 and CA3 hippocampal subfields of AD

To identify common AD-related pathway and biological process changes between sexes and sex-specific alterations in each subfield, we conducted enrichment analysis using the KEGG and GO-BP libraries in Metascape. The focus was specifically on the DEGs obtained from the differential expression analysis between: (a) M-controls vs. F-controls (b) F-AD vs. F-controls, (c) M-AD vs. M- controls and (d) M-AD vs. F-AD. Cancer pathways were excluded from the enriched KEGG pathway results to enhance the specificity of the analysis within the context of the nervous system.

#### 3.2.1 CA1 hippocampal subfield

##### 3.2.1.1 Enrichment analysis results of M-controls vs. F-controls

The enrichment analysis of the top 400 DEGs (top 200 upregulated and top 200 downregulated) between M-controls vs. F-controls identified 28 statistically significant KEGG pathways, grouped into 14 clusters (**Supplementary Table S10**). Notably, the results included the Alzheimer’s disease KEGG cluster, which contained various pathways associated with neurodegenerative diseases (NDs) such as Alzheimer’s Disease (AD), Parkinson’s Disease (PD), Huntington’s Disease, and Amyotrophic lateral sclerosis (ALS). Further analysis revealed that these pathways are upregulated, with the AD pathway containing *BACE1*, a gene found among the top 200 upregulated DEGs.

##### 3.2.1.2 Enrichment analysis results of F-AD vs. F-controls and M-AD vs. M-controls

The analysis uncovered 27 statistically significant KEGG pathways for the CA1 hippocampal subfield in F-AD vs. F-controls (**Supplementary Table S11**). The top three scoring pathway clusters were: (i) Epstein-Barr virus infection (hsa05169), (ii) Focal adhesion (hsa04510), and (iii) Arrhythmogenic right ventricular cardiomyopathy (hsa05412) (**Supplementary Table S11**). In contrast, for the CA1 region in M-AD vs. M-controls, the analysis identified 28 enriched KEGG pathways (**Supplementary Table S12**). The top three scoring pathway clusters were: (i) Estrogen signaling pathway (hsa04915), (ii) Circadian entrainment (hsa04713), and (iii) Wnt signaling pathway (hsa04310) (**Supplementary Table S12**).

**Figure 4:**
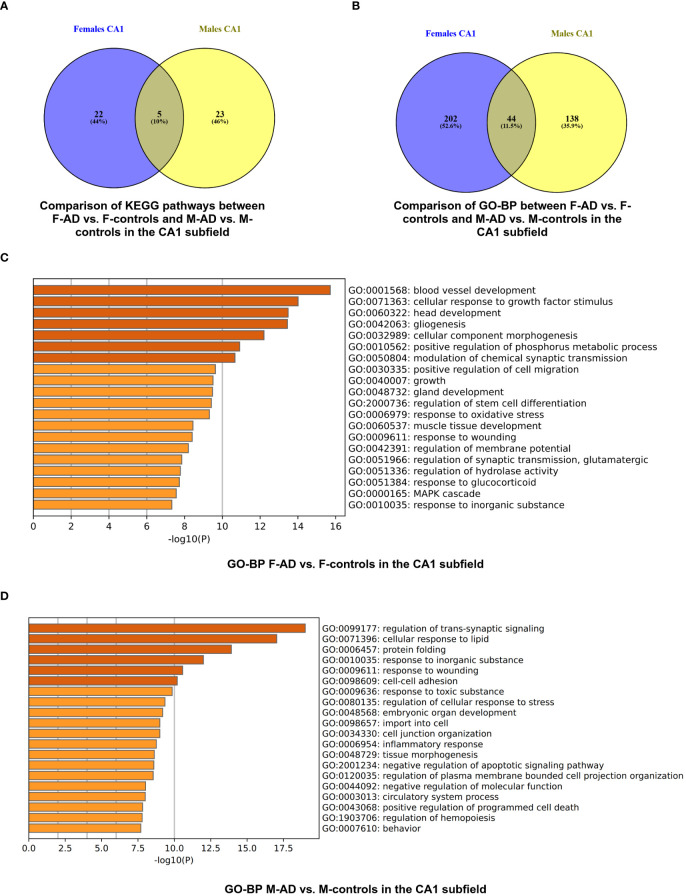
Functional Enrichment Analysis results for CA1 Hippocampal Subfield of GSE29378 dataset with Metascape: **(A)** Comparison between the enriched KEGG pathway terms in F-AD vs. F-controls and M-AD vs. M-controls **(B)** Comparison between the enriched GO-BP terms in F-AD vs. F- controls and M-AD vs. M-controls, **(C)** GO-BP for F-AD vs. F-controls, and **(D)** GO-BP for M-AD vs. M-controls. The intensity of the orange color corresponds to the p-value of the term. A darker shade indicates a more significant p-value.

Comparison between the enriched KEGG pathway terms in F-AD vs. F- controls and M-AD vs. M- controls from the CA1 region indicated five common pathways between males and females with AD: (i) IL-17 signaling pathway (hsa04657), (ii) Fluid shear stress and atherosclerosis (hsa05418), (iii) Oxytocin signaling pathway (hsa04921), (v) MAPK signaling pathway (hsa04010), and (vi) Glutamatergic synapse (hsa04724) (**Figure 4A**). Additionally, sex-specific pathways were identified, with AD male-specific pathways in the CA1 subfield including Estrogen signaling pathway (hsa04915), Toxoplasmosis (hsa05145), Malaria (hsa05144), Insulin secretion (hsa04911), and GABAergic synapse (hsa04727). AD female-specific pathways encompassed Epstein-Barr virus infection (hsa05169), HIF-1 signaling pathway (hsa04066), Calcium signaling pathway (hsa04020) and Dopaminergic synapse (hsa04728).

Subsequent analysis involved a separate enrichment analysis of the top 200 upregulated and top 200 downregulated DEGs to determine the regulatory direction of the enriched pathways. For the Estrogen signaling pathway (hsa04915) in M-AD vs. M-controls, findings revealed both upregulation and downregulation of the pathway in the CA1 subfield. Further analysis using the top 100 upregulated and top 100 downregulated DEGs found between M-AD vs. M-controls suggested that the upregulation of estrogen signaling pathway is even evident with more stringent criteria, while is downregulation was not evident. This suggests that, in M-AD vs. M-controls, the upregulation of the Estrogen signaling pathway could potentially be a compensatory response to the neurodegenerative processes associated with the disease. This upregulation may be indicative of the neuroprotective effects linked to estrogen, highlighting a possible adaptive mechanism in response to neurodegeneration. Additionally, it indicated that AD male-specific pathways, Insulin secretion (hsa04911) and GABAergic synapse (hsa04727), are downregulated in the CA1 subfield.

Moreover, the subsequent analysis indicated that the AD female-specific pathways in the CA1 subfield such as Epstein-Barr virus infection (hsa05169), HIF-1 signaling pathway (hsa04066) and Calcium signaling pathway (hsa04020) are upregulated, whereas the Dopaminergic synapse(hsa04728) was shown to be downregulated. Furthermore, it indicated that IL-17 signaling pathway (hsa04657) and Fluid shear stress and atherosclerosis (hsa05418) pathways are upregulated in both males and females with AD, while Glutamatergic synapse (hsa04724) and Oxytocin signaling pathway (hsa04921) are downregulated in both sexes.

The enrichment analysis of the CA1 hippocampal subfield in F-AD vs. F-Controls revealed 246 statistically significant GO-BP (**Supplementary Table S13**). Conversely, for M-AD vs. M-Controls, the analysis identified 182 enriched GO-BP (**Supplementary Table S14**). The comparison of enriched GO-BP terms in F-AD vs. F- controls and M-AD vs. M- controls from the CA1 hippocampal subfield indicated 44 common biological processes between the males and females with AD (**Figure 4B**). These processes include biological phenomena such as the positive regulation of the MAPK cascade, cellular responses to metal and calcium ions, angiogenesis, and the regulation of synaptic plasticity.

The top three scoring pathway clusters in F-AD vs. F-Controls were: (i) blood vessel development (GO:0001568), cellular response to growth factor stimulus (GO:0071363) and (iii) head development (GO:0060322) (**Figure 4C**). The top three scoring pathway clusters in M-AD vs. M- controls were: (i) regulation of trans-synaptic signaling (GO:0099177), (ii) cellular response to lipid (GO:0071396) and (iii) protein folding (GO:0006457) (**Figure 4D**).

##### 3.2.1.3 Enrichment analysis results of M-AD vs. F-AD

The enrichment analysis of the CA1 hippocampal subfield in M-AD vs. F-AD revealed 25 statistically significant KEGG pathways, grouped into 14 clusters (**Supplementary Table S15**). Notably, pathogen-related pathways such as Coronavirus disease - COVID-19, Legionellosis, and Salmonella infection, along with immune-related pathways like Antigen processing and presentation and TNF signaling pathway, were all found to be downregulated in M-AD compared to F-AD.

#### 3.2.2 CA3 hippocampal subfield

##### 3.2.2.1 Enrichment analysis results of M-controls vs. F-controls

The enrichment analysis of the top 400 DEGs between M-controls vs. F-controls for the CA3 hippocampal subfield identified 25 statistically significant KEGG pathways, grouped into 10 clusters (**Supplementary Table S16**). Consistent with the CA1 subregion, these results included pathways associated with NDs such as AD, PD, and ALS, all of which were upregulated. Interestingly, the AD pathway also featured *BACE1* and the *APP* gene, both among the top 200 upregulated DEGs in the CA3 subfield.

##### 3.2.2.2 Enrichment analysis results of F-AD vs. F-controls and M-AD vs. M-controls

**Figure 5:**
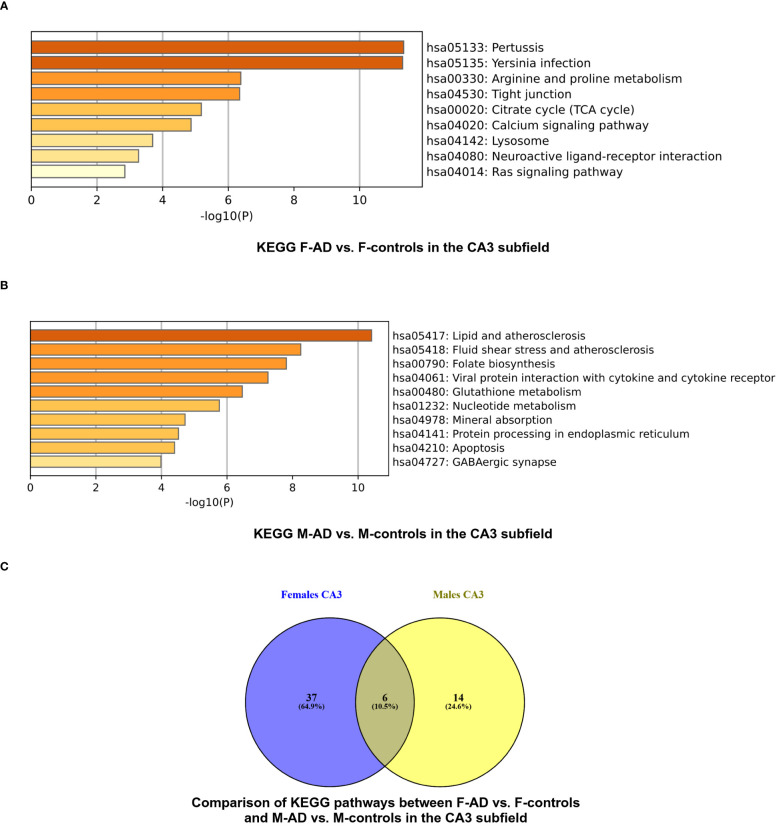
KEGG pathway functional enrichment results for the CA3 Hippocampal Subfield of GSE29378 dataset with Metascape: **(A)** KEGG pathways for F-AD vs. F-controls, **(B)** KEGG pathways for M-AD vs. M-controls, and **(C)** Comparison between the enriched KEGG pathway terms in F-AD vs. F-controls and M-AD vs. M-controls.

The enrichment analysis of the CA3 hippocampal subfield in F-AD vs. F-controls revealed 43 statistically significant KEGG pathways, organized into 9 clusters (**Supplementary Table S17**). The top three scoring pathway clusters were: (i) Pertussis (hsa05133), (ii) Yersinia infection (hsa05135), and (iii) Arginine and proline metabolism (hsa00330) (**Figure 5A**). Conversely, for the CA3 region in M-AD vs. M-controls, the analysis identified 20 enriched KEGG pathways, organized into 10 clusters (**Supplementary Table S18**). The top three scoring pathway clusters were: (i) Lipid and atherosclerosis (hsa05417), (ii) Fluid shear stress and atherosclerosis (hsa05418), and (iii) Folate biosynthesis (hsa00790) (**Figure 5B**).

Comparison between the enriched KEGG pathway terms in F-AD vs. F-controls and M-AD vs. M- controls from the CA3 hippocampal subfield indicated six common pathways between males and females: (i) IL-17 signaling pathway (hsa04657), (ii) MAPK signaling pathway (hsa04010), (iii) Lipid and atherosclerosis (hsa05417), (iv) TNF signaling pathway (hsa04668), (v) Fluid shear stress and atherosclerosis, and (vi) Apoptosis (hsa04210) (**Figure 5C**). Additionally, sex-specific pathways were identified, with the Estrogen signaling pathway (hsa04915) and GABAergic synapse pathway (hsa04727) being specific to M-AD vs. M-controls, and the Calcium signaling pathway (hsa04020) and Dopaminergic synapse pathway (hsa04728) being specific to F-AD vs. F-controls in the CA3 subfield.

Subsequent separate enrichment analysis of the top 200 upregulated and top 200 downregulated DEGs to determine the regulatory direction of the enriched pathways indicated that IL-17 signaling pathway (hsa04657), TNF signaling pathway (hsa04668) and Apoptosis (hsa04210) are upregulated in both sexes. Additionally, it indicated that the AD female-specific pathways in the CA3 subfield Calcium signaling pathway (hsa04020) and Dopaminergic synapse pathway (hsa04728) are downregulated. Moreover, in the CA3 subfield, male-specific pathways in AD showed an upregulation of the Estrogen signaling pathway (hsa04915) and a downregulation of the GABAergic synapse pathway (hsa04727).

**Figure 6:**
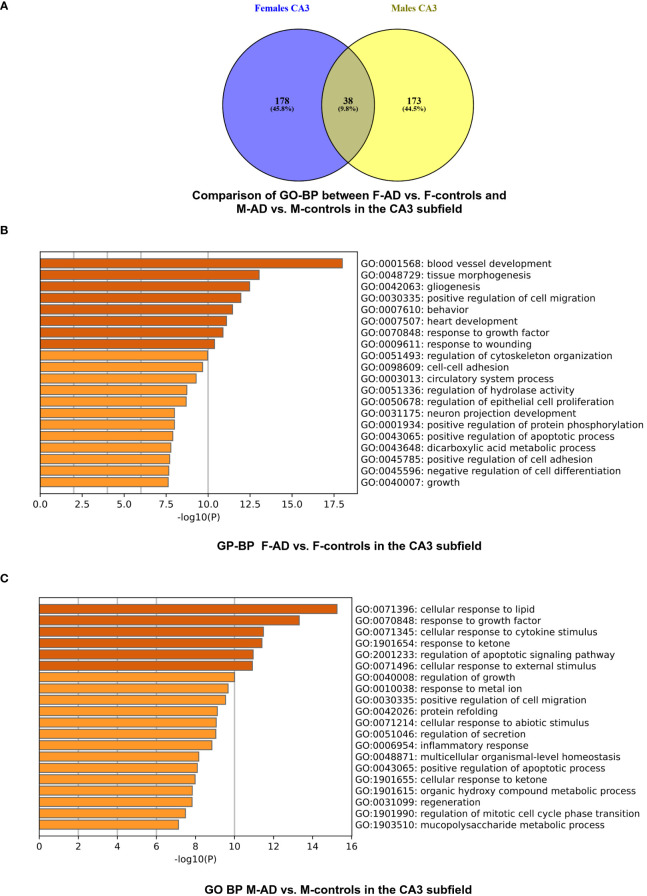
GO-BP functional enrichment results for CA3 Hippocampal Subfield of GSE29378 dataset with Metascape **(A)** Comparison between the GO-BP in F-AD vs. F-controls and M-AD vs. M-controls, **(B)** GO-BP for F-AD vs. F-controls, and **(C)** GO-BP for M-AD vs. M-controls.

The enrichment analysis of the CA3 hippocampal subfield in F-AD vs. F-controls revealed 216 statistically significant GO-BP (**Supplementary Table S19**). Conversely, for M-AD vs. M-controls, the analysis identified 211 enriched GO-BP (**Supplementary Table S20**). Comparison of the enriched GO-BP terms between F-AD vs. F-controls and M-AD vs. M-controls from the CA3 hippocampal subfield indicated 38 common biological processes between males and females (**Figure 6A**). These processes include biological phenomena such as positive regulation of angiogenesis, cellular response to tumor necrosis factor, cellular response to metal ion and cellular response to cytokine stimulus.

The top three scoring pathway clusters in F-AD vs. F-controls were: (i) blood vessel development (GO:0001568), (ii) tissue morphogenesis (GO:0048729) and (iii) gliogenesis (GO:0042063) (**Figure 6B**). The top three scoring pathway clusters M-AD vs. M-controls were: (i) cellular response to lipid (GO:0071396), response to growth factor (GO:0070848) and (iii) cellular response to cytokine stimulus (GO:0071345) (**Figure 6C**).

#### 3.2.3 Comparison of enriched KEGG pathways between F-AD vs. F- controls and M-AD vs. M- controls in the CA1 and CA3 hippocampal subfields

**Figure 7:**
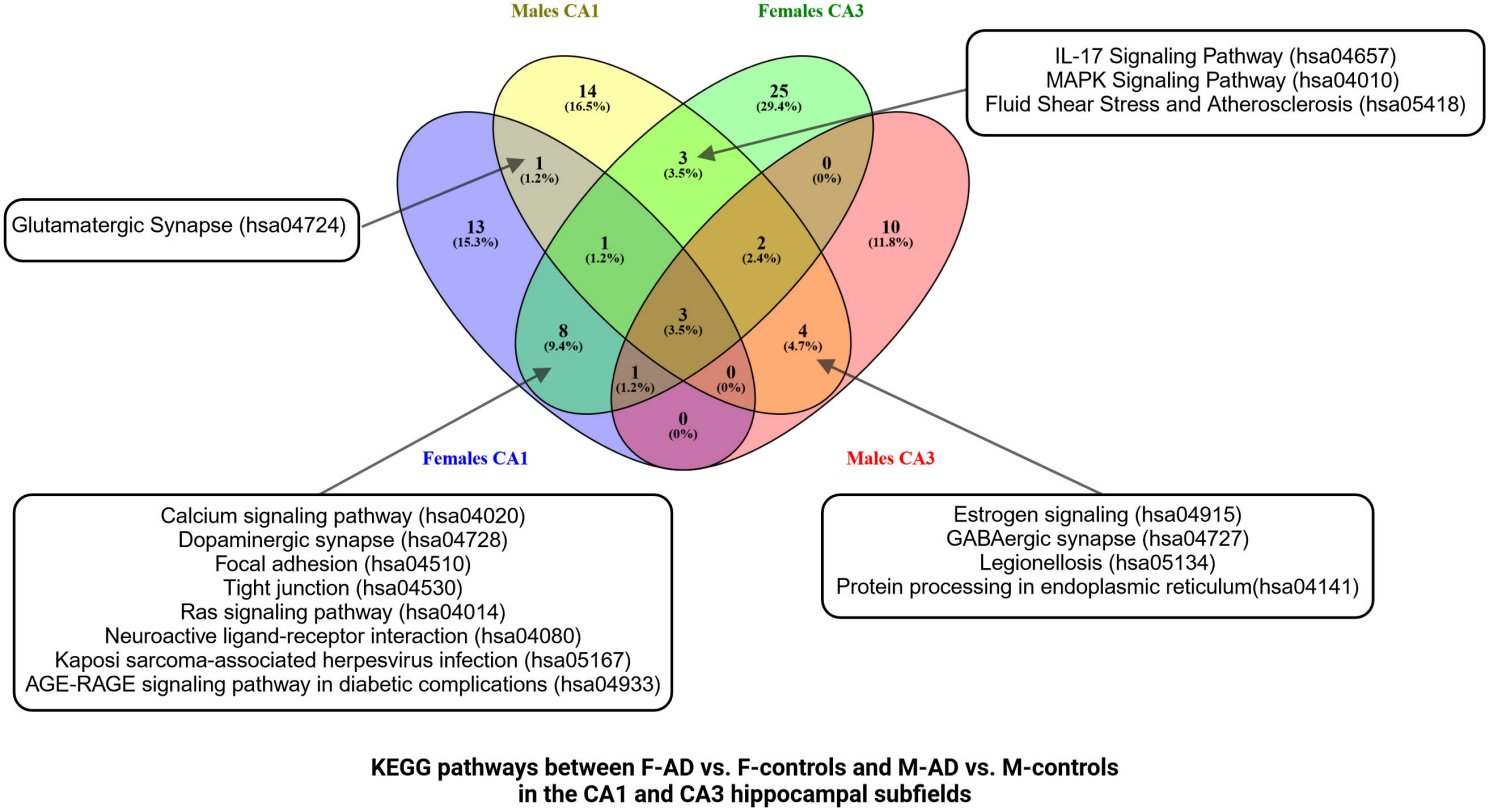
Venn Diagram comparing enriched KEGG pathways between F-AD vs. F-controls and M-AD vs. M- controls in the CA1 and CA3 hippocampal subfields.

Venny comparison of the enriched KEGG pathway terms in the CA1 and CA3 hippocampal subfields between F-AD vs. F- controls and M-AD vs. M-controls, highlighted shared molecular mechanisms in both subfields, as well as subfield-specific pathways (**Figure 7**).

##### 3.2.2.3 Enrichment analysis results of M-AD vs. F-AD

The enrichment analysis of M-AD vs. F-AD in the CA3 hippocampal subfield identified 31 statistically significant KEGG pathways, organized into 13 clusters (**Supplementary Table S21**). In line with the CA1 subfield, the results included pathogen-related pathways like Coronavirus disease - COVID-19. Interestingly, pathways related to GABAergic and Dopaminergic synapses were unique to the CA3 subfield in M-AD compared to F-AD. Subsequent analysis with the top 200 downregulated DEGs confirmed the downregulation of these pathways, but more stringent analysis with the top 100 downregulated DEGs did not show enrichment for these pathways.

### 3.3 Sex-specific transcriptomic differences in the whole hippocampus in AD

#### 3.3.1 DEGs of M-controls vs. F-controls

Through the transcriptome comparison between M-controls vs. F-controls we identified 257 DEGs in the GSE5281 and 294 in the GSE48350 datasets in postmortem hippocampal brain tissue (**Supplementary Tables S22, S23**) respectively.

#### 3.3.2 Comparison of F-AD vs. F-controls and M-AD vs. M-controls

Comparing F-AD vs. F controls, we found 5280 DEGs in the GSE5281 dataset and 7904 DEGs in the GSE48350 dataset (**Supplementary Tables S24, S25** **).** Similarly, comparing M-AD vs. M-controls we identified 5949 DEGs in the GSE5281 and 2644 DEGs in the GSE48350 datasets in postmortem hippocampal brain tissue (**Supplementary Tables S26, S27**).

**Figure 8:**
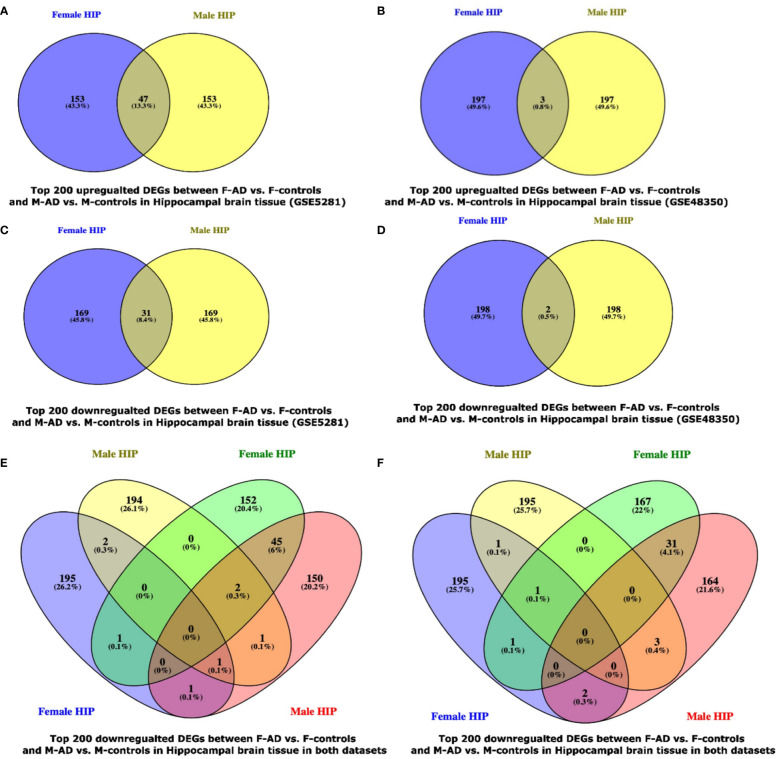
Venn Diagram indicating the common and exclusive DEGs found between F-AD vs. F-controls and M-AD vs. M-controls in postmortem hippocampal brain tissue from the GSE29378 and GSE48350 Datasets: **(A)** top 200 upregulated DEGs between F-AD vs. F-controls and M-AD vs. M-controls in Hippocampal brain tissue in the GSE5281 dataset, **(B)** top 200 upregulated DEGs between F-AD vs. F-controls and M-AD vs. M-controls in Hippocampal brain tissue in the GSE48350 dataset, **(C)** top 200 downregulated DEGs between F-AD vs. F-controls and M-AD vs. M-controls in Hippocampal brain tissue in the GSE5281 dataset, **(D)** top 200 downregulated DEGs between F-AD vs. F-controls and M-AD vs. M-controls in Hippocampal brain tissue in the GSE48350 dataset, **(E)** top 200 upregulated DEGs between F-AD vs. F-controls and M-AD vs. M-controls in Hippocampal brain tissue in both datasets, and **(F)** top 200 downregulated DEGs between F-AD vs. F-controls and M-AD vs. M-controls in Hippocampal brain tissue in both datasets.

The Venny (https://www.biotools.fr/misc/venny) tool was used to compare the top 200 upregulated DEGs in F-AD vs. F-controls and M-AD vs. M-controls in the GSE5281 dataset, revealing 47 common DEGs, while 3 common DEGs were identified in the GSE48350 dataset (see **Figures 8A, B**). Additionally, the comparison between the top 200 downregulated DEGs in F-AD vs. F-controls and M-AD vs. M-controls in the hippocampal brain tissue revealed 31 and 2 DEGs, respectively, in the two datasets mentioned above (see **Figures 8C, D**). Moreover, of the top 200 upregulated (see **Figure 8E**) and downregulated (see **Figure 8F**) DEGs revealed no common DEGs between the four groups.

**Figure 9:**
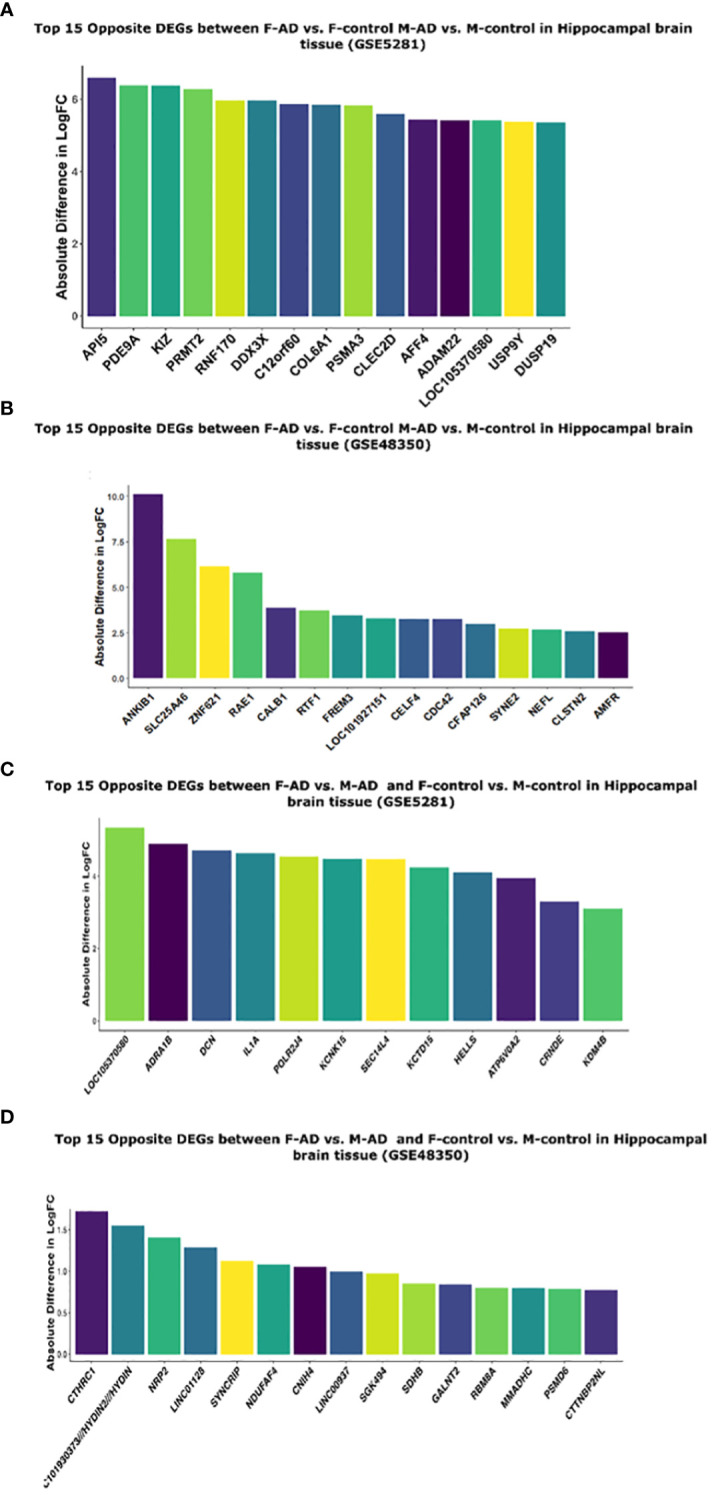
Top 15 opposite DEGs based on absolute difference in logFC between: **(A)** F-AD vs. F-controls and M-AD vs. M-controls in Hippocampal brain tissue (GSE5281), **(B)** F-AD vs. F-controls and M-AD vs. M-controls in Hippocampal brain tissue (GSE48350), **(C)** F-AD vs. M-AD and F-control vs. M-controls in Hippocampal brain tissue (GSE5281), and **(D)** F-AD vs. M-AD and F-control vs. M-controls in Hippocampal brain tissue (GSE48350).

Moreover, to further explore the difference between F-AD vs. F-controls and M-AD vs. M-controls we identified the opposite DEGs found between the two comparison groups in each dataset separately for postmortem hippocampal brain tissue based on the absolute difference in logFC. This approach allowed for the isolation of the top 15 opposite DEGs between F-AD vs. F-controls and M-AD vs. M-controls. In the GSE5281 hippocampal dataset, the top 15 opposite DEGs are: *API5, PDE9A, KIZ, PRMT2, RNF170, DDX3X, C12orf60, COL6A1, PSMA3, CLEC2D, AFF4, ADAM22, LOC105370580, USP9Y, DUSP19* (**Figure 9A**). Similarly, the top 15 opposite DEGs for the GSE48350 dataset include: *ANKIB1, SLC25A46, ZNF621, RAE1, CALBA, RTF1, FREM3, LOC101927151, CELF4, CDC42, CFAP126, SYNE2, NEFL, CLSTN2, AMFR* (**Figure 9B**).

#### 3.3.3 DEGs of M-AD vs. F-AD

Direct comparison between M-AD vs. F-AD revealed 1939 DEGs in the GSE5281 dataset and 1301 DEGs in the GSE48350 dataset (**Supplementary Tables S28, S29**) respectively.

#### 3.3.4 Comparison between M-controls vs. F-controls and M-AD vs. F-AD based on absolute difference in logFC

Moreover, to further explore the difference between M-controls vs. F-controls and M-AD vs. F-AD we identified opposite DEGs in the GSE5281 and GSE48350 datasets for postmortem hippocampal brain tissue based on the absolute difference in logFC. Thus, enabling us to isolate the top 15 opposite DEGs between the groups of interest. In the GSE5281 dataset, these DEGs include, *LOC10530580, ADRA1B, DCN, IL1A, POLR2J4, KCNK15, SEC14L4, KCTD16, HELLS, ATP6V0A2, CRNDE, KDM4B* (**Figure 9C**). The latter for the GSE48350 DEGs include, *CTHRC1, LOC10193373//HYDIN2//HYDIN, NRP2, LINCO112B, SYNCRIP, NDUFAF4, CNIH4, LINC00937, SGK494, SDHB, GALNT2, RBM8A, NMADHC, PSMD6, CTTNBP2NL* (**Figure 9D**).

### 3.4 KEGG and GO-BP enrichment analysis of shared DEGs and sex-specific patterns in whole hippocampal region of AD

Similarly to section 3.2, enrichment analysis was also performed on the top DEGs obtained from the GSE5281 (22, 23) and GSE48350 (24, 25) datasets.

#### 3.4.1 Whole hippocampus KEGG enriched results

##### 3.4.1.1 Enrichment analysis results of M-controls vs. F-controls

The enrichment analysis of the top 400 DEGs (top 200 upregulated and top 200 downregulated) for the GSE5281 dataset between M-controls and F-controls identified 10 statistically significant KEGG pathways, grouped into 7 clusters (**Supplementary Table S30**). Mainly consisting of signaling pathways such as PI3K-Akt, Thyroid hormone, Hippo, Calcium and TGF-beta signaling pathways. Further analysis revealed both upregulation and downregulation of the PI3K-Akt and Calcium signaling pathways, while Hippo and TGF-beta pathways were upregulated.

Similarly, enrichment analysis was also performed for the top 400 DEGs (top 200 upregulated and top 200 downregulated) for the GSE48350 dataset between M-controls and F-controls, revealing 14 statistically significant KEGG pathways, grouped into 6 clusters (**Supplementary Table S31**). Pathways related to cellular function were observed, including the proteasome, mRNA surveillance pathway, spliceosome, citrate cycle (TCA cycle), and mitophagy. Additionally, pathways associated with neurodegeneration such as AD, PD, and ALS were identified. The viral infectious pathways herpes simplex virus 1 infection was also identified. Further analysis revealed downregulation of the citrate cycle pathway.

##### 3.4.1.2 Enrichment analysis results of F-AD vs. F-controls and M-AD vs. M-controls

The KEGG enrichment analysis of the GSE5281 dataset revealed 32 statistically significant pathways grouped into 6 clusters for F-AD vs. F-controls. The three top scoring pathways were: (i) Antigen processing and presentation (hsa04612), (ii) Bacterial invasion of epithelial cells (hsa05100) and (iii) Prion disease (hsa05020), (**Supplementary Table S32**). Conversely, for M-AD vs. M-controls in the same dataset, 34 significant pathways and 13 pathway clusters were identified. The top three scoring pathways were: (i) Morphine addiction (hsa05032), (ii) AGE-RAGE signaling pathway in diabetic complications (hsa04933) and (iii) cAMP signaling (hsa04024) for the GSE5281 dataset (**Supplementary Table S33**).

In the GSE48350 dataset, contrasting results were observed. F-AD vs. F-controls revealed 40 statistically significant pathways grouped into 17 clusters. The top three scoring pathways were: (i) GABAergic synapse (hsa04727), (ii) Rheumatoid arthritis (hsa05323), and (iii) Synaptic vesicle cycle (hsa04721) (**Supplementary Table S34**). Conversely, for M-AD vs. M-controls, 13 statistically significant pathways grouped into 8 clusters were identified. The top three scoring KEGG pathways were: (i) Viral life cycle-HIV (hsa03250), (ii) Bacterial invasion of epithelial cells (hsa05100) and (iii) RNA degradation (has03018) (**Supplementary Table S35**).

Subsequent analysis involved separate enrichment analysis of the top 200 upregulated and top 200 downregulated DEGs for each dataset to determine the regulatory direction of the enriched pathways. In the GSE5281 dataset, antigen processing and presentation were identified to be downregulated in F-AD compared to F-controls, while several signaling pathways, including Apelin and calcium signaling pathways, were shown to be upregulated in M-AD vs. M-controls. Moreover, in M-AD vs. M-controls, Circadian entrainment (hsa04713), along with Axon guidance (hsa04360) and the Sphingolipid signaling pathway (hsa04071) were downregulated.

In contrast, in the GSE48350 dataset, several neuron-related pathways, including GABAergic synapse, Glutamatergic synapse (hsa04724), Synaptic vesicle cycle (hsa04721), and Long-term depression (hsa04730), were upregulated in F-AD vs. F-controls. Additionally, the TGF-beta signaling pathway (hsa04350) was observed to be downregulated in F-AD compared to F-controls. For the comparison of M-AD vs. M-controls in the GSE48350 dataset, the downregulated pathways included RNA degradation (hsa03018) and regulation of actin cytoskeleton (hsa04810).

Functional enrichment analysis of whole Hippocampal brain tissue for both the GSE5281 and GSE48350 datasets was also performed using the GO-BP library. In F-AD vs. F-controls, 180 and 208 statistically significant GO-BP terms were revealed for the GSE5281 and GSE48350 datasets, respectively (**Supplementary Tables S36, S37**). Conversely, 134 and 249 statistically significant GO-BP terms for M-AD vs. M-controls were revealed for the GSE5281 and GSE48350 datasets, respectively (**Supplementary Tables S38, 39**).

**Figure 10:**
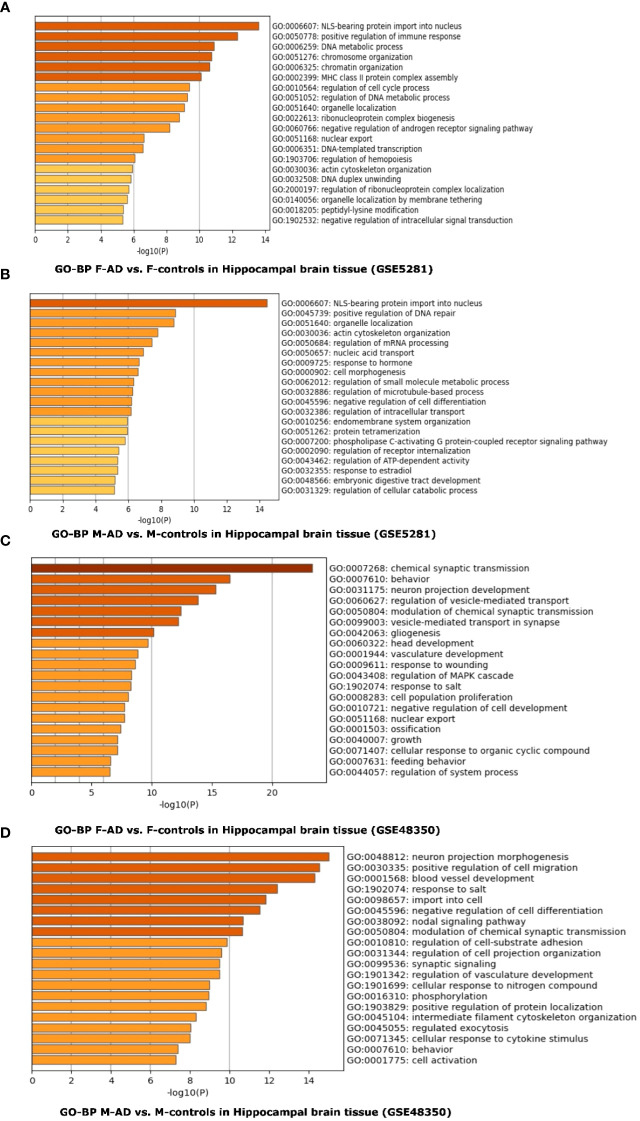
Functional Enrichment Analysis results for whole Hippocampal brain tissue of GSE5281 and GSE48350 datasets with Metascape: **(A)** Comparison between the enriched GO-BP terms in F-AD vs. F-controls in GSE5281 dataset, **(B)** Comparison between the enriched GO-BP terms in M-AD vs. M-controls in GSE5281 dataset, **(C)** Comparison between the enriched GO-BP terms in F-AD vs. F-controls in GSE48350 dataset and **(D)** Comparison between the enriched GO-BP terms in M-AD vs. M-controls in GSE48350 dataset.

The top three scoring pathways in the F-AD vs. F-controls for GSE5281 were: (i) NLS-bearing protein import into nucleus (GO:0006607), (ii) positive regulation of immune response (GO:0050778) and DNA metabolic process (GO:0006259) (**Figure 10A**). Conversely, for M-AD vs. M-controls in GSE5281 the top pathways were: (i) NLS-bearing protein import into nucleus (GO:0006607), (ii) positive regulation of DNA repair (GO: 0045739) and (iii) organelle localization (GO:0051640) (**Figure 10B**).

In contrast, for F-AD vs. F-controls in the GSE48350 dataset, the top three pathways were related to the nervous system: (i) Chemical synaptic transmission (GO:0007268), (ii) Behavior (GO:0007610) and (ii) Neuron projection development (GO:0031175) (**Figure 10C**). Conversely, the GO-BP terms of (i) neuron projection morphogenesis (GO:0048812), positive regulation of cell migration (GO:0030335), and blood vessel development (GO:0001568) were identified for M-AD vs. M-control in the GSE48350 dataset (**Figure 10D**).

##### 3.4.1.3 Enrichment analysis results of M-AD vs. F-AD

KEGG enrichment for M-AD vs. F-AD in GSE5281 and GSE48350 datasets can be found in (**Supplementary Tables S40, 41**) respectively. The direct comparison between M-AD vs. F-AD in the GSE5281 dataset revealed 34 significantly enriched KEGG pathways between males and females with AD, including the Calcium signaling pathway and GABAergic synapse pathways. In contrast, for the GSE48350 dataset, it revealed 7 enriched KEGG pathways.

### 3.5 Investigation of the interaction of *ESR1*, *ESR2* and *GPER1* with AD susceptibility genes

**Figure 11:**
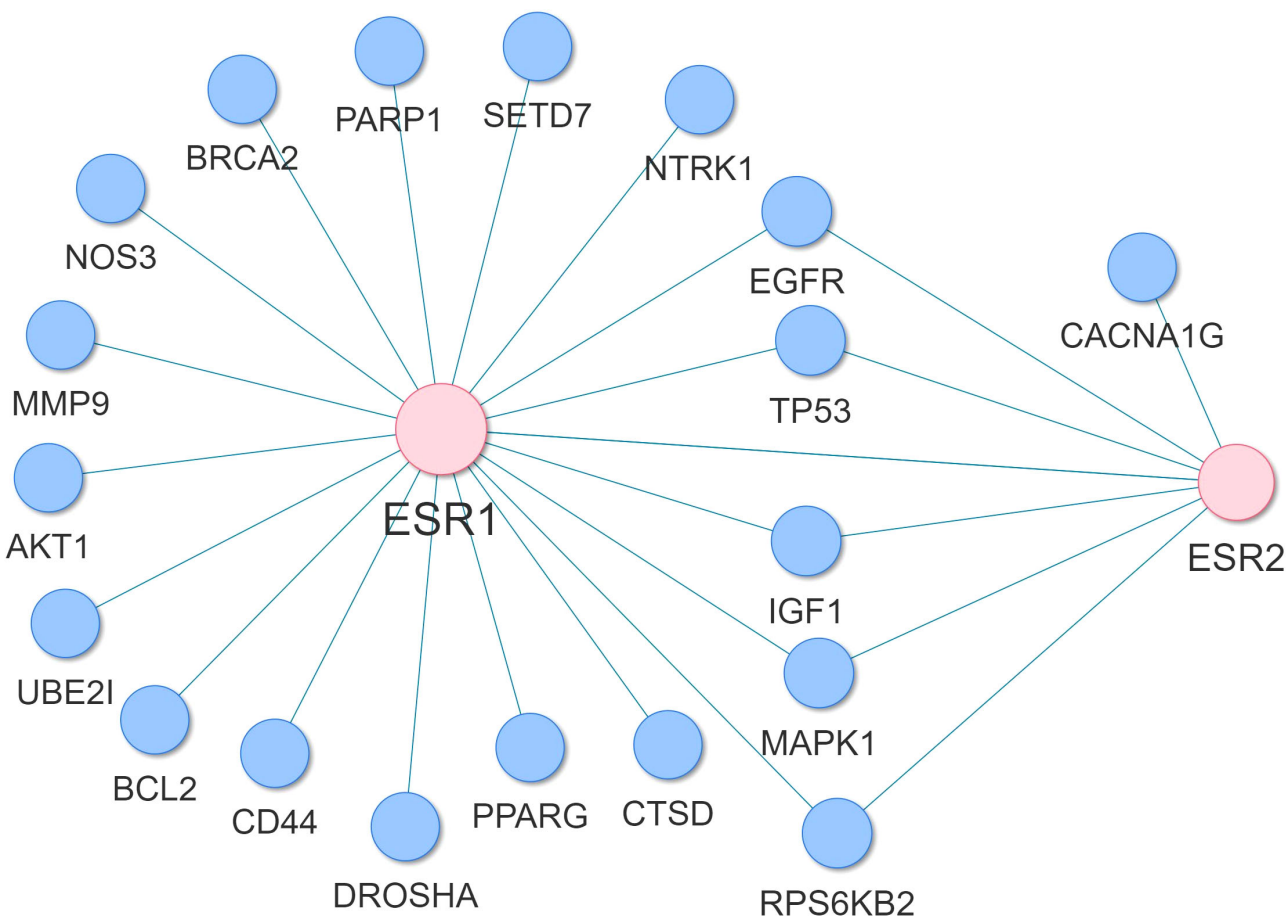
Subnetwork of the AD variant-variant PPI network indicating the AD susceptibility genes that interact with *ESR1* and *ESR2*.

Through the reconstruction of the AD variant-variant PPI network, comprising genetic susceptibility genes associated with AD, we have identified potential susceptibility genes that interact with *ESR1, ESR2* and *GPER1*. *ESR1* and *ESR2* genes are the primary receptors targeted by hormone replacement drugs in menopause, while, *GPER1*, also expressed in the brain, may modulate rapid estrogen processes like calcium fluxes (32). In the reconstructed AD variant-variant PPI network, we observed that the ESR1 gene interacts with several susceptibility genes, including *UBE2I, TP53, SETD7, RPS6KB2, PPARG, PARP1, NTRK1, NOS3, MMP9, MAPK1, IGF1, ESR2, EGFR, DROSHA, CTSD, CD44, BRCA2, BCL2*, and *AKT1* (**Figure 11**). Additionally, the *ESR2* gene interacts with susceptibility genes such as *CACNA1G, EGFR, ESR1, IGF1, MAPK1, RPS6KB2*, and *TP53* within the network (**Figure 11**). Moreover, the *GPER1* gene interacts with the *ESR1*, *ESR2* and *EGFR* AD susceptibility genes. These interactions suggest that these AD susceptibility genes may be functionally connected to the estrogen receptor pathways. This provides insight into potential molecular mechanisms by which hormonal influences, particularly estrogen signaling, intersect with the genetic factors associated with AD risk.

## 4 Discussion

The hippocampus, crucial for memory and learning, is early impacted by AD (7), with women being twice as likely as men to develop the disease and experiencing greater cognitive decline (8, 9). Our study focused on elucidating molecular mechanisms influencing AD susceptibility in women, particularly in the hippocampus and CA1 and CA3 subfields. We also investigated the interaction of AD susceptibility genes with *ESR1* and *ESR2*, key targets of menopause hormone replacement drugs, as well as *GPER1.* Our findings underscore significant molecular distinctions between sexes, revealing sex-specific alterations in signaling pathways associated with AD. In M-AD vs. M-controls, the GABAergic synapse was downregulated, and the Estrogen signaling pathway was upregulated in both CA1 and CA3 subfields, contrary to F-AD vs. F-controls. Conversely, F-AD vs. F-controls exhibited downregulation of the Dopaminergic synapse in both subfields, while the Calcium signaling pathway showed mixed regulation. Additionally, the IL-17 Signaling Pathway was commonly upregulated in both sexes in both CA1 and CA3 subfields. Moreover, when comparing M-AD vs. F-AD, we observed a small upregulation of the *ESR1* gene in the CA1 subfield of males with AD compared to females with AD. Furthermore, direct comparison of M-AD vs. F-AD included pathogen-related pathways like Coronavirus disease - COVID-19. Additionally, potential susceptibility genes interacting with estrogen receptors *ESR1* and *ESR2* were identified, including *MAPK1, IGF1, AKT1, TP53*, and *CD44*.

Comparison of the enriched KEGG pathway results of the top 400 DEGs of F-AD vs. F-controls in the GSE5281 dataset revealed various pathways related to the immune (Antigen processing and presentation) and nervous systems (Prion disease and Oxytocin signaling pathway but also cellular organization and function (Adherence junction, Gap junction). Antigen processing and presentation was identified to be downregulated in F-AD compared to F-controls. Antigen processing and presentation by microglia represents a critical aspect of the brain’s immune surveillance and response. Microglia, the brain’s immune effectors, play a crucial role for maintaining CNS homeostasis by collaborating with astrocytes and T-cells (33). Chronic neuroinflammation and age-related senescence activate microglia; however, inappropriate processing of misfolded proteins via the lysosomal pathway allows for the spreading of toxic protein constituents to the healthy neurons (33). Microglia process and present self-antigen (β-amyloid and phosphorylated Tau) to infiltrated CD4^+^ T-cells VIA MHCI/II molecules. Following this process, the microglial phenotype can be altered from a proactive M1 to a neuroprotective M2 type that corresponds to tissue remodeling and homeostasis (33). Thus, the downregulation of antigen processing and presentation in F-AD vs. F-controls suggests a potential impairment in microglial-mediated immune surveillance, critical for detecting and clearing abnormal protein aggregates characteristic of AD pathology.

Circadian entrainment was found to be downregulated in M-AD vs. M-controls in the GSE5281 dataset. Sleep-related pathological symptomatology usually accompanies AD, and circadian dysfunction occurs in both healthy aging and in age-related diseases. Previous data suggests that disruption of the circadian rhythm is more pronounced in AD, and therefore, sleep disturbances and circadian rhythm alternations maybe useful indicators for AD disease development (34). Specifically, sleep alternations include loss of slow-wave sleep (SWS) and REM stage sleep. Previous studies suggest that REM phase sleep remains unaffected during the early stages of AD but starts to decrease during the late stages (34).

Regarding the GSE48350 dataset, pathways related to the nervous system (GABAergic synapse, Glutamatergic synapse, Long-term depression and Synaptic vesicle cycle) were upregulated in F-AD vs. F-controls. Evidence has shown that GABAergic synapses contribute to AD progression by disrupting neuronal communication required for information processing (35). Synapse transmission involves receptors activating and binding to pre-synaptic and post-synaptic sites; moreover, synaptic transmission depends on long-term potential (LTP) and long-term depression which are mechanisms essential for learning and memory (35). One study found that the temporal cortex of AD patients demonstrated decreased levels of GABA and Glutamate, resulting in synaptic and neurotransmission dysfunction. Furthermore, decreased GABA neurotransmitters were associated with aging, demonstrating that AD patients with amyloid plaques have GABA synapse dysfunction (35). GABAergic synapse function is influenced by multiple key components including environmental factors, lifestyle-related factors (diet, physical activity and sleep), biological factors (age and sex), and molecular factors (Aβ, p-tau, APOE, astrocytes and microglia). Notably, research on gender difference in AD are limited; however, studies have shown dementia to be the fifth and eighth leading cause of death in women and men, respectively, in the United States (35).

The TGF-beta signaling pathway was found to be downregulated in F-AD vs. F-controls in the GSE48350 dataset. TGF-beta, including isoforms TGF-β1, -2, and -3 are pleiotropic cytokines with neuroprotective, and immunoregulation functions (36, 37). The TGF-β type II receptor (TβRII) is mainly expressed by neurons, and its levels are reduced in human AD brain, correlating with pathological hallmarks of the disease (37). Numerous studies suggest that a decline in TGF-β1 signaling is closely associated with increased deposition of Aβ plaques and NFTs in AD. In comparison to healthy aged individuals, decreased levels of TGF-β1, were observed in plasma and serum of AD patients, along with a reduced level of TGF-β1 released from peripheral blood cells. Furthermore, patients with mild cognitive impairment (MCI) have been identified to be at a greater risk of AD development due to a decrease of anti-inflammatory TGF-β and increased production of proinflammatory TNFα (37).

To further explore sex-specific differences in the hippocampus of individuals with AD, we analyzed the GSE29378 dataset, focusing on the CA1 and CA3 subfields. Among the top 200 upregulated DEGs in F-AD vs. F-controls with M-AD vs. M-controls, 16 common genes were found, including *CD163*, primarily expressed on microglia (38). *CD163-*expressing microglia respond to amyloid (39), indicating the presence of specific microglia phenotypes in the CA1 and CA3 subfields acting as a defense against Aβ accumulation in both sexes. *RGS1*, another commonly upregulated DEG, exhibited the highest logFC in both subfields. It plays a role in atherosclerosis, attracting macrophages to atherosclerotic plaques and modulating inflammatory responses (40). Its consistent upregulation across sexes suggests its comparable involvement in the immune response associated with AD. Moreover, *EFHD2*, emerged as the sole common downregulated DEGs among the top 200 downregulated DEGs in both subfields. In AD it co-localizes with tau protein, suggesting a potential role in tau-mediated neurodegeneration (41, 42). Further research is needed to elucidate its exact role and its potential as a therapeutic target.

To identify KEGG pathways associated with the top DEGs, we utilized two complementary enrichment analysis approaches. Initially, we conducted pathway enrichment analysis using all top 400 DEGs (top 200 upregulated and 200 downregulated) together. Subsequently, we performed separate enrichment analyses for the top 200 upregulated and top 200 downregulated DEGs to discern the regulatory direction of the enriched KEGG pathways. Integrating both approaches provides a comprehensive understanding of sex-specific molecular mechanisms in AD. By analyzing all DEGs together, we capture the global dysregulation of the pathways involved, while separate analyses offer insights into the specific molecular alterations driving disease pathology in males and females. This approach overcomes limitations in enrichment analysis, ensuring meaningful signals are not masked.

The enrichment analysis unveiled sex-specific patterns in the CA1 and CA3 hippocampal subfields. Specifically, the GABAergic synapse and Estrogen signaling pathways were enriched in both the CA1 and CA3 subfields in M-AD vs. M-controls but not in F-AD vs. F-controls. Notably, the GABAergic synapse was downregulated, while the Estrogen signaling pathway was upregulated. Gamma-aminobutyric acid (GABA) is the primary inhibitory neurotransmitter in the central nervous system, crucial for synaptic plasticity and excitatory-inhibitory balance in the hippocampus (43). Dysregulation of the GABAergic synapse is implicated in AD, contributing to memory and cognitive deficits, that are distinct from age-related decline (43–46). On the other hand, the Estrogen signaling pathway exerts neuroprotective effects in the hippocampus, by reducing neuroinflammation and enhancing synaptic plasticity (12). It interacts with AD-associated pathways, such as mitogen-activated protein kinase (MAPK) signaling, forming a complex network influencing disease susceptibility (47). Estrogen-mediated neuroprotection against Aβ involves activating the MAPK signaling pathway (48), that was also found enriched in both the CA1 and CA3 hippocampal subfields across sexes.

The upregulation of the Estrogen signaling pathway in M-AD vs. M-controls suggests a compensatory response to neurodegeneration, potentially contributing to distinct cognitive/memory deficits observed in M-AD compared to F-AD. However, the underlying mechanisms of this sex-specific dysregulation remain unclear. Nonetheless, we can speculate on potential contributing factors. Estrogen receptors, such as *ESR1*, are known to play complex roles in neuroprotection, influencing synaptic plasticity, anti-inflammatory responses, and antioxidant processes (49). The observed upregulation of *ESR1* between M-AD vs. F-AD in the CA1 subfield may indicate a sex-specific modulation of estrogen receptor expression, affecting its neuroprotective effects. Moreover, estrogen interacts with pathways involved in Aβ processing and tau hyperphosphorylation, suggesting its relevance in AD pathology. These findings highlight the significance of sex-specific differences in AD and suggest a distinct role of estrogen signaling in males versus females with AD. Further research is required to understand the underlying mechanisms and their implications for AD treatment.

Conversely, F-AD vs. F-controls exhibited enrichment of the Dopaminergic synapse and Calcium signaling pathways in both the CA1 and CA3 subfields, contrasting with M-AD vs. M-controls. The Dopaminergic synapse was downregulated in both subfields, while the Calcium signaling pathway showed mixed regulation, being upregulated in CA1 but downregulated in CA3. Calcium dysregulation is implicated in AD pathology (50, 51), triggering processes leading to Aβ plaques and NFTs formation (52, 53). Aβ accumulation elevates intracellular Ca2+, impacting neuronal metabolism, and promoting apoptosis (51). Abnormal tau protein aggregation, influenced by calcium-mediated changes, contributes to synaptic loss and neuronal degeneration (51). Maintaining calcium homeostasis is crucial for normal neuronal function, and disruptions in this balance contribute to AD pathogenesis (51). Further research is needed to explore the specific mechanisms underlying these sex-specific differences in calcium dysregulation in AD.

Dopamine is critical for memory functions in the hippocampus (54, 55), and alternations in its neurotransmission are linked to cognitive changes in aging (56) and AD (57). While studies on dopamine receptor levels in AD show mixed results, meta-analyses indicate decreased dopaminergic neurotransmitters in AD (58). Our fundings show downregulation of the Dopaminergic Synapse pathway, exclusively in women with AD, potentially explaining previous discrepancies. This suggests that dopamine-related changes could contribute to AD susceptibility and progression, particularly in women.

Deficits in dopaminergic signaling in AD are associated with neuropsychiatric symptoms (NPS) and mood regulation (59–61). NPS are prevalent in AD, so reduced dopamine levels may contribute to the onset of depressive symptoms (60). Therefore, the downregulation of the Dopaminergic Synapse pathway, observed in both the CA1 and CA3 hippocampal subfields in women with AD, may contribute to depressive symptoms. Conversely, men with AD show downregulation in the GABAergic synapse. Abnormalities in GABAergic signaling also contribute to depressive symptoms (62). Our findings suggest potential sex-specific mechanisms underlying NPS in AD. These sex-specific neurotransmitter differences may inform tailored treatments for NPS symptoms in AD, considering variations between males and females.

A common enriched pathway between M-AD vs. M-controls and F-AD vs. F-controls was the IL-17 Signaling Pathway, upregulated in both subfields. Interleukin 17 (IL-17) is a potent pro-inflammatory cytokine implicated in NDs like AD, exacerbating neuroinflammation by promoting immune cell infiltration into the brain (63). Interestingly, IL-17 inhibits neurogenesis in the adult hippocampus, while its absence enhances neurogenesis and improves synaptic function (64). Our findings suggest the IL-17 Signaling Pathway as a potential therapeutic for both sexes.

In the CA1 subfield, F-AD vs. F-controls show pathways related to viral infections (Epstein-Barr virus and Kaposi sarcoma-associated herpesvirus), while M-AD vs. M-controls exhibit pathways related to bacterial and parasitic infectious (Toxoplasmosis, Malaria, and Chagas disease). Such distinctions may influence sex-specific susceptibility and progression. In the CA3 subfield, both F-AD vs. F-controls and M-AD vs. M-controls showed enrichment in pathways linked to both viral and bacterial infections, reflecting a different pattern. Additionally, the direct comparison between M-AD vs. F-AD revealed pathogen-related pathways such as Coronavirus disease - COVID-19 in both subfields, suggesting a potential link between viral infections and AD pathology. Environmental risk factors, including viruses and bacteria, may contribute to AD pathology (65, 66). Previous studies also link SARS-CoV-2 infection and members of the *Herpesviridae* Family to AD development through modulation of AD-related processes via virus-host protein-protein interactions (67, 68). Women generally exhibit stronger immune responses, making them more effective at fighting infections (69), but potentially increasing susceptibility to immune-mediated diseases (70). Sex hormones, particularly estrogen in women, modulate immune cell function (71, 72). While estrogen boost humoral immunity, androgens and progestogens predominantly induce immunosuppressive effects (69). Further research is needed to determine if specific infections, such as COVID-19, increase AD risk in women compared to men, and to explore sex differences in the underlying mechanisms.

The comparison between M-controls vs. F-controls revealed upregulation in pathways associated with AD and other NDs in both hippocampal subfields. This indicates differences in gene expression between healthy males with normal cognitive and functional examinations compared to healthy females (normomics). This suggests potential sex-specific differences in AD susceptibility or progression. Notably, *BACE1*, a key enzyme linked to amyloid-beta production in AD pathology, was found to be upregulated in M-controls compared to F-controls. This observation suggests that males may have a higher baseline expression of *BACE1*, potentially leading to increased amyloid-beta production and plaque formation, characteristic of AD. Therefore, the observed differences in *BACE1* expression patterns between males and females’ controls may contribute to sex-specific vulnerabilities or protective mechanisms in AD pathogenesis. This intriguing finding underscores the complex interplay between sex, age, and disease mechanisms in AD pathogenesis, especially considering the typically higher susceptibility of women to AD despite similar ages between the groups. However, caution is needed due to the dataset’s disproportionate gender distribution. Further research is necessary to elucidate these differences and their implications for AD and other NDs. Through reconstruction of the AD variant-variant PPI network, potential susceptibility genes that interacting with the estrogen receptors *ESR1* and *ESR2* were identified, including *MAPK1, IGF1, AKT1, TP53* and *CD44*. Additionally, the *GPER1* gene was found to interact with the *ESR1*, *ESR2* and *EGFR* AD susceptibility genes. These findings suggest a link between the identified susceptibility genes and estrogen receptors, necessitating further research to elucidate specific functional consequences. Investigation into whether genetic polymorphisms in these genes render women more vulnerable to hormonal fluctuations and their impact on AD risk is crucial for personalized medicine strategies. Of interest is the interplay between estrogen, Insulin-like Growth Factor 1 (IGF1), AKT, and MAPK signaling pathways, known for their neuroprotective effects. Evidence indicates their synergistic action in promoting neuroprotection (73–76), suggesting potential for hormone replacement drugs to reduce AD susceptibility in women through these pathways.

To our knowledge, our study represents the first application of bioinformatics methodologies for the analysis of publicly available mRNA transcriptomic data, with the explicit aim of investigating sex differences and similarities in the hippocampus and its subfields. Despite inherent limitations stemming from the relatively small number of both female and male groups within the available hippocampal datasets, as well as the lack of information on HRT status for the female groups, which hinders a comprehensive understanding of estrogen signaling pathways, and the unavailability of Braak staging data for all samples further limits our ability to compare gene expression patterns across different stages of AD, representing another constraint in our analysis. However, despite these limitations, our research yields invaluable insights into sex-specific differences in AD susceptibility and progression in the hippocampus and its subfields. The observed sex differences can guide the development of targeted and personalized therapeutic interventions. Tailoring treatments based on sex-specific molecular responses could enhance their effectiveness and reduce sex-based disparities in AD outcomes.

## Data availability statement

The original contributions presented in the study are included in the article/**Supplementary Material.** Further inquiries can be directed to the corresponding author.

## Ethics statement

Ethical approval was not required for the study involving humans in accordance with the local legislation and institutional requirements. Written informed consent to participate in this study was not required from the participants or the participants’ legal guardians/next of kin in accordance with the national legislation and the institutional requirements.

## Author contributions

AO: Data curation, Formal analysis, Funding acquisition, Methodology, Writing – original draft, Writing – review & editing. CC: Data curation, Formal analysis, Methodology, Writing – original draft, Writing – review & editing. EZ-P: Resources, Supervision, Writing – original draft, Writing – review & editing. PZ: Funding acquisition, Resources, Supervision, Writing – original draft, Writing – review & editing. PG: Conceptualization, Funding acquisition, Investigation, Methodology, Resources, Supervision, Writing – original draft, Writing – review & editing.

## References

[1] Mendez MF. Early-onset Alzheimer disease and its variants. Contin Lifelong Learn Neurol. (2019) 25:34–51. doi: 10.1212/CON.0000000000000687 PMC6538053 30707186

[2] Rujeedawa T Félez EC Clare ICH Fortea J Strydom A Rebillat AS. The clinical and neuropathological features of sporadic (Late-onset) and genetic forms of alzheimer’s disease. J Clin Med. (2021) 10:4582. doi: 10.3390/jcm10194582 34640600 PMC8509365

[3] Eid A Mhatre I Richardson JR. Gene-environment interactions in Alzheimer’s disease: A potential path to precision medicine. Pharmacol Ther. (2019) 199:173–87. doi: 10.1016/j.pharmthera.2019.03.005 PMC6827882 30877021

[4] O’Brien RJ Wong PC. Amyloid precursor protein processing and alzheimer’s disease. Annu Rev Neurosci. (2011) 34:185–204. doi: 10.1146/annurev-neuro-061010-113613 21456963 PMC3174086

[5] Chen GF Xu TH Yan Y Zhou YR Jiang Y Melcher K. Amyloid beta: Structure, biology and structure-based therapeutic development. Acta Pharmacol Sin. (2017) 38:1205–35. doi: 10.1038/aps.2017.28 PMC5589967 28713158

[6] Iqbal K Liu F Gong C-X Grundke-Iqbal I. Tau in alzheimer disease and related tauopathies. Curr Alzheimer Res. (2010) 7:656–64. doi: 10.2174/156720510793611592 PMC3090074 20678074

[7] Braak H Braak E. Staging of alzheimer-related cortical destruction. Int Psychogeriatrics. (1997) 9:257–61. doi: 10.1017/S1041610297004973 9447446

[8] Snyder HM Asthana S Bain L Brinton R Craft S Dubal DB. Sex biology contributions to vulnerability to Alzheimer’s disease: A think tank convened by the Women’s Alzheimer’s Research Initiative. Alzheimer’s Dement. (2016) 12:1186–96. doi: 10.1016/j.jalz.2016.08.004 PMC10341380 27692800

[9] Laws KR Irvine K Gale TM. Sex differences in cognitive impairment in Alzheimer’s disease. World J Psychiatry. (2016) 6:54. doi: 10.5498/wjp.v6.i1.54 27014598 PMC4804268

[10] Sohn D Shpanskaya K Lucas JE Petrella JR Saykin AJ Tanzi RE. Sex Differences in Cognitive Decline in Subjects with High Likelihood of Mild Cognitive Impairment due to Alzheimer’s disease. Sci Rep. (2018) 8:7490. doi: 10.1038/s41598-018-25377-w 29748598 PMC5945611

[11] Burke SL Hu T Fava NM Li T Rodriguez MJ Schuldiner KL. Sex differences in the development of mild cognitive impairment and probable Alzheimer’s disease as predicted by hippocampal volume or white matter hyperintensities. J Women Aging. (2019) 31:140–64. doi: 10.1080/08952841.2018.1419476 PMC6039284 29319430

[12] Brann DW Dhandapani K Wakade C Mahesh VB Khan MM. Neurotrophic and neuroprotective actions of estrogen: Basic mechanisms and clinical implications. Steroids. (2007) 72:381–405. doi: 10.1016/j.steroids.2007.02.003 17379265 PMC2048656

[13] Klein SL Flanagan KL. Sex differences in immune responses. Nat Rev Immunol. (2016) 16:626–38. doi: 10.1038/nri.2016.90 27546235

[14] Zandi PP Carlson MC Plassman BL Welsh-Bohmer KA Mayer LS Steffens DC. Hormone replacement therapy and incidence of Alzheimer disease in older women: The Cache County Study. Jama. (2002) 288:2123–9. doi: 10.1001/jama.288.17.2123 12413371

[15] Carroll JC Rosario ER Chang L Stanczyk FZ Oddo S LaFerla FM. Progesterone and estrogen regulate Alzheimer-like neuropathology in female 3xTg-AD mice. J Neurosci. (2007) 27:13357–65. doi: 10.1523/JNEUROSCI.2718-07.2007 PMC6673397 18045930

[16] Coughlan GT Betthauser TJ Boyle R Koscik RL Klinger HM Chibnik LB. Association of age at menopause and hormone therapy use with tau and β-amyloid positron emission tomography. JAMA Neurol. (2023) 80:462–73. doi: 10.1001/jamaneurol.2023.0455 PMC10071399 37010830

[17] Ratnakumar A Zimmerman SE Jordan BA Mar JC. Estrogen activates Alzheimer’s disease genes. Alzheimer’s Dement Transl Res Clin Interv. (2019) 5:906–17. doi: 10.1016/j.trci.2019.09.004 PMC6926344 31890855

[18] Xing Y Jia JP Ji XJ Tian T. Estrogen associated gene polymorphisms and their interactions in the progress of Alzheimer’s disease. Prog Neurobiol. (2013) 111:53–74. doi: 10.1016/j.pneurobio.2013.09.006 24096044

[19] Pirskanen M Hiltunen M Mannermaa A Helisalmi S Lehtovirta M Hänninen T. Estrogen receptor beta gene variants are associated with increased risk of Alzheimer’s disease in women. Eur J Hum Genet. (2005) 13:1000–6. doi: 10.1038/sj.ejhg.5201447 15944651

[20] Miller JA Woltjer RL Goodenbour JM Horvath S Geschwind DH. Genes and pathways underlying regional and cell type changes in Alzheimer’s disease. Genome Med. (2013) 5:48. doi: 10.1186/gm452 23705665 PMC3706780

[21] Yao Z Dong H Zhu J Du L Luo Y Liu Q. Age-related decline in hippocampal tyrosine phosphatase PTPRO is a mechanistic factor in chemotherapy-related cognitive impairment. JCI Insight. (2023) 8:e166306. doi: 10.1172/jci.insight.166306 37485875 PMC10443805

[22] Liang WS Dunckley T Beach TG Grover A Mastroeni D Walker DG. Gene expression profiles in anatomically and functionally distinct regions of the normal aged human brain. Physiol Genomics. (2007) 28:311–22. doi: 10.1152/physiolgenomics.00208.2006 PMC2259385 17077275

[23] Liang WS Dunckley T Beach TG Grover A Mastroeni D Ramsey K. Altered neuronal gene expression in brain regions differentially affected by Alzheimer’s disease: A reference data set. Physiol Genomics. (2008) 33:240–56. doi: 10.1152/physiolgenomics.00242.2007 PMC2826117 18270320

[24] Berchtold NC Cribbs DH Coleman PD Rogers J Head E Kim R. Gene expression changes in the course of normal brain aging are sexually dimorphic. Proc Natl Acad Sci U.S.A. (2008) 105:15605–10. doi: 10.1073/pnas.0806883105 PMC2563070 18832152

[25] Berchtold NC Coleman PD Cribbs DH Rogers J Gillen DL Cotman CW. Synaptic genes are extensively downregulated across multiple brain regions in normal human aging and Alzheimer’s disease. Neurobiol Aging. (2013) 34:1653–61. doi: 10.1016/j.neurobiolaging.2012.11.024 PMC4022280 23273601

[26] Ritchie ME Phipson B Wu D Hu Y Law CW Shi W. Limma powers differential expression analyses for RNA-sequencing and microarray studies. Nucleic Acids Res. (2015) 43:e47. doi: 10.1093/nar/gkv007 25605792 PMC4402510

[27] Zhou Y Zhou B Pache L Chang M Khodabakhshi AH Tanaseichuk O. Metascape provides a biologist-oriented resource for the analysis of systems-level datasets. Nat Commun. (2019) 10:1523. doi: 10.1038/s41467-019-09234-6 30944313 PMC6447622

[28] Piñero J Ramírez-Anguita JM Saüch-Pitarch J Ronzano F Centeno E Sanz F. The DisGeNET knowledge platform for disease genomics: 2019 update. Nucleic Acids Res. (2020) 48:D845–55. doi: 10.1093/nar/gkz1021 PMC7145631 31680165

[29] Doncheva NT Morris JH Holze H Kirsch R Nastou KC Cuesta-Astroz Y. Cytoscape stringApp 2.0: analysis and visualization of heterogeneous biological networks. J Proteome Res. (2023) 22:637–46. doi: 10.1021/acs.jproteome.2c00651 PMC9904289 36512705

[30] Meysman P Titeca K Eyckerman S Tavernier J Goethals B Martens L. Protein complex analysis: From raw protein lists to protein interaction networks. Mass Spectrom Rev. (2017) 36:600–14. doi: 10.1002/mas.21485 26709718

[31] Bozhilova LV Whitmore AV Wray J Reinert G Deane CM. Measuring rank robustness in scored protein interaction networks. BMC Bioinf. (2019) 20:446. doi: 10.1186/s12859-019-3036-6 PMC6714100 31462221

[32] Prossnitz ER Barton M. Signaling, physiological functions and clinical relevance of the G protein-coupled estrogen receptor GPER. Prostaglandins Other Lipid Mediat. (2009) 89:89–97. doi: 10.1016/j.prostaglandins.2009.05.001 PMC2740807 19442754

[33] Das R Chinnathambi S. Microglial priming of antigen presentation and adaptive stimulation in Alzheimer’s disease. Cell Mol Life Sci. (2019) 76:3681–94. doi: 10.1007/s00018-019-03132-2 PMC11105582 31093687

[34] Homolak J Mudrovčić M Vukić B Toljan K. Circadian rhythm and alzheimer’s disease. Med Sci (Basel Switzerland). (2018) 6:52. doi: 10.3390/medsci6030052 PMC6164904 29933646

[35] Rivera J Sharma B Torres MM Kumar S. Factors affecting the GABAergic synapse function in Alzheimer’s disease: Focus on microRNAs. Ageing Res Rev. (2023) 92:102123. doi: 10.1016/j.arr.2023.102123 37967653

[36] Tesseur I Zou K Esposito L Bard F Berber E Van Can J. Deficiency in neuronal TGF-β signaling promotes neurodegeneration and Alzheimer’s pathology. J Clin Invest. (2006) 116:3060–9. doi: 10.1172/JCI27341 PMC1626127 17080199

[37] Kapoor M Chinnathambi S. TGF-β1 signalling in Alzheimer’s pathology and cytoskeletal reorganization: a specialized Tau perspective. J Neuroinflamm. (2023) 20:72. doi: 10.1186/s12974-023-02751-8 PMC10012507 36915196

[38] Garton T Keep RF Hua Y Xi G. CD163, a hemoglobin/haptoglobin scavenger receptor, after intracerebral hemorrhage: functions in microglia/macrophages versus neurons. Transl Stroke Res. (2017) 8:612–6. doi: 10.1007/s12975-017-0535-5 28386733

[39] Nguyen AT Wang K Hu G Wang X Miao Z Azevedo JA. APOE and TREM2 regulate amyloid-responsive microglia in Alzheimer’s disease. Acta Neuropathol. (2020) 140:477–93. doi: 10.1007/s00401-020-02200-3 PMC7520051 32840654

[40] Patel J Mcneill E Douglas G Hale AB De Bono J Lee R. RGS1 regulates myeloid cell accumulation in atherosclerosis and aortic aneurysm rupture through altered chemokine signalling. Nat Commun. (2015) 6:6614. doi: 10.1038/ncomms7614 25782711 PMC4374153

[41] Ferrer-Acosta Y Rodríguez-Cruz EN Orange F De Jesús-Cortés H Madera B Vaquer-Alicea J. EFhd2 is a novel amyloid protein associated with pathological tau in Alzheimer’s disease. J Neurochem. (2013) 125:921–31. doi: 10.1111/jnc.12155 PMC3676478 23331044

[42] Vega IE. EFhd2, a protein linked to Alzheimer’s disease and other neurological disorders. Front Neurosci. (2016) 10:150. doi: 10.3389/fnins.2016.00150 27064956 PMC4814571

[43] Xu Y Zhao M Han Y Zhang H. GABAergic inhibitory interneuron deficits in alzheimer’s disease: implications for treatment. Front Neurosci. (2020) 14:660. doi: 10.3389/fnins.2020.00660 32714136 PMC7344222

[44] Huang Y Mucke L. Alzheimer mechanisms and therapeutic strategies. Cell. (2012) 148:1204–22. doi: 10.1016/j.cell.2012.02.040 PMC3319071 22424230

[45] Govindpani K Guzmán BCF Vinnakota C Waldvogel HJ Faull RL Kwakowsky A. Towards a better understanding of GABAergic remodeling in alzheimer’s disease. Int J Mol Sci. (2017) 18:1813. doi: 10.3390/ijms18081813 28825683 PMC5578199

[46] Selkoe DJ. Early network dysfunction in Alzheimer’s disease. Science (80-). (2019) 365:540–1. doi: 10.1126/science.aay5188 31395769

[47] Koszegi Z Cheong RY. Targeting the non-classical estrogen pathway in neurodegenerative diseases and brain injury disorders. Front Endocrinol (Lausanne). (2022) 13:999236. doi: 10.3389/fendo.2022.999236 36187099 PMC9521328

[48] Fitzpatrick JL Mize AL Wade CB Harris JA Shapiro RA Dorsa DM. Estrogen-mediated neuroprotection against β-amyloid toxicity requires expression of estrogen receptor α or β and activation of the MAPK pathway. J Neurochem. (2002) 82:674–82. doi: 10.1046/j.1471-4159.2002.01000.x 12153491

[49] Chen P Li B Ou-Yang L. Role of estrogen receptors in health and disease. Front Endocrinol (Lausanne). (2022) 13:839005. doi: 10.3389/fendo.2022.839005 36060947 PMC9433670

[50] Joshi M Joshi S Khambete M Degani M. Role of calcium dysregulation in Alzheimer’s disease and its therapeutic implications. Chem Biol Drug Des. (2023) 101:453–68. doi: 10.1111/cbdd.14175 36373976

[51] Ge M Zhang J Chen S Huang Y Chen W He L. Role of calcium homeostasis in alzheimer’s disease. Neuropsychiatr Dis Treat. (2022) 18:487–98. doi: 10.2147/NDT.S350939 PMC8901263 35264851

[52] Khachaturian ZS. Calcium hypothesis of Alzheimer’s disease and brain aging. Ann N Y Acad Sci. (1994) 747:1–11. doi: 10.1111/j.1749-6632.1994.tb44398.x 7847664

[53] Popugaeva E Pchitskaya E Bezprozvanny I. Dysregulation of neuronal calcium homeostasis in Alzheimer’s disease – A therapeutic opportunity? Biochem Biophys Res Commun. (2017) 483:998–1004. doi: 10.1016/j.bbrc.2016.09.053 27641664 PMC5303663

[54] Heath FC Jurkus R Bast T Pezze MA Lee JLC Voigt JP. Dopamine D1-like receptor signalling in the hippocampus and amygdala modulates the acquisition of contextual fear conditioning. Psychopharmacol (Berl). (2015) 232:2619–29. doi: 10.1007/s00213-015-3897-y PMC4480849 25743759

[55] Atherton LA Dupret D Mellor JR. Memory trace replay: The shaping of memory consolidation by neuromodulation. Trends Neurosci. (2015) 38:560–70. doi: 10.1016/j.tins.2015.07.004 PMC4712256 26275935

[56] Bäckman L Lindenberger U Li SC Nyberg L. Linking cognitive aging to alterations in dopamine neurotransmitter functioning: Recent data and future avenues. Neurosci Biobehav Rev. (2010) 34:670–7. doi: 10.1016/j.neubiorev.2009.12.008 20026186

[57] Martorana A Koch G. Is dopamine involved in Alzheimer’s disease? Front Aging Neurosci. (2014) 6:252. doi: 10.3389/fnagi.2014.00252 25309431 PMC4174765

[58] Pan X Kaminga AC Wen SW Wu X Acheampong K Liu A. Dopamine and dopamine receptors in Alzheimer’s disease: A systematic review and network meta-analysis. Front Aging Neurosci. (2019) 10:175. doi: 10.3389/fnagi.2019.00175 PMC6637734 31354471

[59] D’Amelio M Puglisi-Allegra S Mercuri N. The role of dopaminergic midbrain in Alzheimer’s disease: Translating basic science into clinical practice. Pharmacol Res. (2018) 130:414–9. doi: 10.1016/j.phrs.2018.01.016 29391234

[60] Chen Y Dang M Zhang Z. Brain mechanisms underlying neuropsychiatric symptoms in Alzheimer’s disease: a systematic review of symptom-general and –specific lesion patterns. Mol Neurodegener. (2021) 16:38. doi: 10.1186/s13024-021-00456-1 34099005 PMC8186099

[61] Campbell S MacQueen G. The role of the hippocampus in the pathophysiology of major depression. J Psychiatry Neurosci. (2004) 29:417–26. PMC524959 15644983

[62] Luscher B Shen Q Sahir N. The GABAergic deficit hypothesis of major depressive disorder. Mol Psychiatry. (2011) 16:383–406. doi: 10.1038/mp.2010.120 21079608 PMC3412149

[63] Ye X Chen J Pan J Wu Q Wang Y Lu M. Interleukin-17 promotes the infiltration of CD8+ T cells into the brain in a mouse model for alzheimer’s disease. Immunol Invest. (2023) 52:135–53. doi: 10.1080/08820139.2022.2136525 36394561

[64] Liu Q Xin W He P Turner D Yin J Gan Y. Interleukin-17 inhibits Adult hippocampal neurogenesis. Sci Rep. (2014) 4:7554. doi: 10.1038/srep07554 25523081 PMC4271266

[65] Sochocka M Zwolińska K Leszek J. The infectious etiology of alzheimer’s disease. Curr Neuropharmacol. (2017) 15:996–1009. doi: 10.2174/1570159x15666170313122937 PMC5652018 28294067

[66] Maheshwari P Eslick GD. Bacterial infection and Alzheimer’s disease: A meta-analysis. J Alzheimer’s Dis. (2015) 43:957–66. doi: 10.3233/JAD-140621 25182736

[67] Onisiforou A Spyrou GM. Systems bioinformatics reveals possible relationship between COVID-19 and the development of neurological diseases and neuropsychiatric disorders. Viruses. (2022) 14:2270. doi: 10.3390/v14102270 36298824 PMC9611753

[68] Onisiforou A Zanos P. From viral infections to alzheimer’s disease: unveiling the mechanistic links through systems bioinformatics. bioRxiv. (2023) 2023:12. doi: 10.1101/2023.12.05.570187 PMC11385591 39255398

[69] Sciarra F Campolo F Franceschini E Carlomagno F Venneri MA. Gender-specific impact of sex hormones on the immune system. Int J Mol Sci. (2023) 24:6302. doi: 10.3390/ijms24076302 37047274 PMC10094624

[70] Ngo ST Steyn FJ McCombe PA. Gender differences in autoimmune disease. Front Neuroendocrinol. (2014) 35:347–69. doi: 10.1016/j.yfrne.2014.04.004 24793874

[71] Ortona E Pierdominici M Rider V. Editorial: Sex hormones and gender differences in immune responses. Front Immunol. (2019) 10:1076. doi: 10.3389/fimmu.2019.01076 31156632 PMC6530401

[72] Shepherd R Cheung AS Pang K Saffery R Novakovic B. Sexual dimorphism in innate immunity: the role of sex hormones and epigenetics. Front Immunol. (2021) 11:604000. doi: 10.3389/fimmu.2020.604000 33584674 PMC7873844

[73] Garcia-Segura LM Arevalo MA Azcoitia I. Interactions of estradiol and insulin-like growth factor-I signalling in the nervous system: New advances. Prog Brain Res. (2010) 181:251–72. doi: 10.1016/S0079-6123(08)81014-X 20478442

[74] Jover-Mengual T Zukin RS Etgen AM. MAPK signaling is critical to estradiol protection of CA1 neurons in global ischemia. Endocrinology. (2007) 148:1131–43. doi: 10.1210/en.2006-1137 PMC2528200 17138646

[75] Singer CA Figueroa-Masot XA Batchelor RH Dorsa DM. The mitogen-activated protein kinase pathway mediates estrogen neuroprotection after glutamate toxicity in primary cortical neurons. J Neurosci. (1999) 19:2455–63. doi: 10.1523/jneurosci.19-07-02455.1999 PMC6786088 10087060

[76] Quesada A Lee BY Micevych PE. PI3 kinase/Akt activation mediates estrogen and IGF-1 nigral DA neuronal neuroprotection against a unilateral rat model of Parkinson’s disease. Dev Neurobiol. (2008) 68:632–44. doi: 10.1002/dneu.20609 PMC2667142 18278798

